# Limitations and safety aspects related to the use of bacteriophages in food production

**DOI:** 10.1093/femsre/fuag002

**Published:** 2026-01-20

**Authors:** Angelika Roth, Charles M A P Franz, Stefan Hertwig, Thomas Holzhauser, Christian Hertel, Hans-Ulrich Humpf, Karl-Heinz Engel, Uwe Schwarzenbolz, Oliver Schlüter, Henry Jäger, Kemal Aganovic, Volker Heinz

**Affiliations:** Leibniz Research Centre for Working Environment and Human Factors (IfADo), D-44139 Dortmund, Germany; Max Rubner-Institut (MRI), D-24103 Kiel, Germany; German Federal Institute for Risk Assessment (BfR), D-10589 Berlin, Germany; Division of Allergology, Paul-Ehrlich-Institut (PEI), D-63225 Langen, Germany; DIL German Institute of Food Technologies e.V., D-49610 Quakenbrück, Germany; Institute of Food Chemistry, University of Münster, D-48140 Münster, Germany; Technical University of Munich (TUM), D-85350 Freising, Germany; Technische Universität Dresden (TUD), D-01062 Dresden, Germany; Leibniz Institute for Agricultural Engineering and Bioeconomy (ATB), D-14469 Potsdam, Germany; University of Natural Resources and Life Sciences (BOKU), AT-1180 Wien, Austria; DIL German Institute of Food Technologies e.V., D-49610 Quakenbrück, Germany; Institute of Food Quality and Food Safety, University of Veterinary Medicine, D-30599 Hannover, Germany; DIL German Institute of Food Technologies e.V., D-49610 Quakenbrück, Germany

**Keywords:** Bacteriophages, phage cocktails, biocontrol, biopreservation, food safety, food production

## Abstract

Bacteriophages are considered to have great potential as naturally occurring, antimicrobial agents for use in food production. Phages are ubiquitous in nature and can be isolated from almost all habitats. This review outlines the possibilities, as well as the limitations of their use in food production. Applications of phages in the food sector are described and the limitations of their use, as well as potential risks, are discussed. Approaches for a possible classification as either processing aid or food additive are considered, and the current status of their use in and outside the EU is presented. Finally, the need for research to close identified knowledge gaps is highlighted.

## Teaser

Bacteriophages can be used as antimicrobial agents and contribute to food safety; however, their range of application and effectiveness are limited, their regulatory classification is not yet conclusively clarified, and certain safety aspects are still debated.

## Introduction

Bacteriophages (short “phages”) are viruses that infect bacteria. They are ubiquitous, with global estimates ranging from 10^31^ to 10^32^ phages. They occur in high numbers in soil (1.5 × 10^8^g^–1^ of agricultural soil), water (10^6^ to 10^9^mL^–1^ of seawater and 7 × 10^6^ to 1.5 × 10^7^mL^–1^ of fresh water), and also in food (Emond and Moineau 2007, Połaska and Sokołowska 2019, Vikram et al. 2021). Phages influence natural bacterial communities and help maintain their balance (Naureen et al. 2020). In the dairy industry, naturally occurring phages can disrupt fermentation cultures and cause production losses (Połaska and Sokołowska 2019). The rising incidence of food-borne infections, including those caused by *Listeria monocytogenes, Campylobacter* spp., Shiga toxin-producing *Escherichia coli*, and *Salmonella* spp. (EFSA 2022) as well as food poisoning caused by, for example, *Staphylococcus aureus* (Hennekinne et al. 2012), *Bacillus cereus* (Jovanovic et al. 2021), and various *Clostridium* species (Wells-Bennik et al. 2016) has increased interest in phages due to their potential as tools for enhancing food safety. At the same time, consumer scepticism toward chemical preservatives has grown, driving demand for naturally occurring antimicrobial agents. Phages offer advantages over conventional preservation techniques because they can selectively target and reduce human-pathogens as well as spoilage-causing bacteria. As biological entities without their own metabolism, they do not affect the organoleptic and quality characteristics of food and can be applied particularly to raw or fresh products such as fruits and vegetables or “ready-to-eat” (RTE) foods consumed without further processing (Hudson et al. 2005, Hagens and Loessner 2010, Cooper 2016, Jagannathan et al. 2022). They can withstand the stress and physicochemical conditions of food processing very well (Jayamanne and Foddai 2025); however, their effectiveness depends on the host bacteria maintaining at least a basic level of metabolic activity (Bryan et al. 2016). Since the naturally occurring phage reservoir in the environment also adapts to changing bacterial populations, it is assumed that effective virulent phages can be constantly isolated from the environment to inactivate newly emerging strains. In addition, phages continuously adapt to the altered defence mechanisms of the host bacteria (Koskella and Brockhurst 2014, Mattila et al. 2015), so that their long-term use is not compromised by the emergence of permanently phage-resistant bacteria.

However, limitations and possible risks of using phages in the food sector have also been addressed (Greer 2005, Hagens and Loessner 2010, Kazi and Annapure 2016, de Melo et al. 2018, Lewis and Hill 2020, Garvey 2022). It is often criticized that there is no guarantee that the phages used will sufficiently inactivate the bacterial species in question. This may be due to their narrow host range, the high diversity of strains and serotypes of the target species, mutated phages with altered host specificity, or the emergence of phage-resistant bacteria. Furthermore, a possible interaction between acquired phage resistance and antibiotic resistance has been suggested as a potential risk of phage use (McGee et al. 2023). There is also concern that phages might be used to replace established, conventional methods needed to ensure microbiological safety, rather than complementing them (EFSA 2016). Phages remaining on food may bias bacterial enrichment and complicate culture-based detection of pathogens, depending on the method used (Fister et al. 2019). The uncontrolled release of large quantities of one or a few phage species into the environment, especially in the case of surface application in animal housing and production facilities, has been considered a potential risk, as there is concern about uncontrolled and possibly critical alteration of existing bacterial communities by the released phages (Fernandez et al. 2018, Vikram et al. 2021). The spread of virulence and antibiotic resistance genes has also been proposed to be a risk of phage applications (Hassan et al. 2021). Negative effects on human gut microbiota or possible allergic reactions due to high intake of phages are also considered potential risks. References addressing the advantages and disadvantages of phage use in the food sector are listed in the Supplementary Table S1.

The objective of this review is to assess the challenges and potential risks associated with phage use in food production (Table 1) based on current scientific data and, where necessary, to identify research needs. The focus will be on the use of phages in food production and on finished food products; however, their application in livestock and crop plants cultivation is also addressed. The classification of phages as inanimate particles with potential biological effects, whether as food additives, processing aids, or agents for reducing bacterial surface contamination on foods of animal origin, will be discussed. This review, however, does not cover the therapeutic use of phages in farm animals. Therapeutic use in humans or as a probiotic is addressed only in relation to the safety of oral phage ingestion.

**Table 1.** Overview on the challenges, limitations, and possible risks of phage use in the food sector that are discussed by [1] (Hagens and Loessner 2010), [2] (Amjad et al. 2024), [3] (Fernandez et al. 2018), [4] (Garvey 2022), [5] (Endersen et al. 2014), [6] (Gildea et al. 2022), [7] (Greer 2005), [5] (Jagannathan et al. 2022), [9] (Vikram et al. 2021), [10] (Kazi and Annapure 2016), [11] (Endersen and Coffey 2020), [12] (Ge et al. 2022), [13] (Cooper 2016), [14] (Fister et al. 2019), [15] (Chaudhary et al. 2024), [16] (de Melo et al. 2018), [17] (Goodridge and Bisha 2011), and [18] (Połaska and Sokołowska 2019) (left column). Proposals for evaluation and consideration the limits and risks, along with potential solutions, are presented (right column).

Challenges/limitations/possible risks of phage use	References	Evaluation of the points listed
		Recommendations for measures
***Efficacy/effectiveness***		
Possible difficulties to transfer results from laboratory/inoculated food items to practice. Food matrix might prevent or strongly influence phage activity.	[1], [2]	Task for applied research. Evaluation needed.
Possible difficulties to adapt lab-scale to large-scale bacteriophage production and purification.	[3], [4]	Responsibility of companies according to good manufacturing practice (GMP).
When should phages be used in the food production chain, and especially at what exact step?	[1]	Task for applied research. Evaluation needed. Education of user.
Narrow host range	[3], [5], [6], [7], [8], [9], [10]	Screening for broad-host-range phages. Use of phage cocktails instead of single phage. Knowledge of contaminating species. Evaluation needed and education of user
Is the target in the special area of application known? Other pathogen species/strains may become dominant on the food to be treated. Food may be contaminated by a mixture of different pathogens that are not targeted by one phage cocktail. Potential emergence of phage-resistant strains or serotypes of a target bacterial species.	[2], [11]	Evaluation of phage cocktails and information on efficacy by the manufacturer. Update and/or replace of phages in cocktails by the manufacturer. Constant checks. Use of different phage cocktails sequentially or in combination. Education of user.
Phage titre is reduced by the conditions/environment of practical application (e.g. food). Sufficient concentration of infectious phage particles? What volume of applied phage solution on solid food, what titre of applied phages? Can the appropriate titre of phages be achieved?	[1], [12]	Possible exclusion of certain foods. Precise instructions for phage application. Task for applied research. Evaluation needed. Education of user.
The phage particles should physically come into contact with all or most of the target bacterial cells, even if the cells/cell colonies are distributed at different, distant points on the surface.	[11]	Possible exclusion of certain foods. Precise instructions for phage application. Task for applied research. Evaluation needed. Education of user.
Is there a minimum host number requirement for phage propagation?	[8]	The required count of host cells cannot be ensured. Propagation is not the intention of phage use, not possible in practice. The phages do not multiply significantly during application or only unintentionally under rare conditions.
Effectiveness in the application depends on metabolically active host cells, suitable pH, temperature, and food matrix. Is there enough information on efficacy of phages on natural contaminated food?	[2], [4], [8], [10], [13], [14]	Possible exclusion of certain foods. Task for applied research. Evaluation needed. Education of user.
May food additives in the products inhibit the efficacy of applied phages?	[13]	Possible exclusion of certain foods. Task for applied research. Evaluation needed.
Reduction lower as with conventional methods	[12]	Generally, the target cells will not be completely eradicated, not all bacterial species/strains/serotypes can be targeted. Phage application is not for substitution of conventional methods.
***General limitations***		
Natural ability of bacteria to develop phage resistance. Coevolution of bacteria and bacteriophages, length of phage use, and repeated use might lead to resistance.	[1], [4], [6], [7], [8], [9], [10], [11], [14], [16], [17], [18]	Long coexistence of phages and metabolic active bacterial cells is not intended, when phages are used on food. May be possible in production facilities and on surfaces. Task for applied research. Evaluation needed.
Exposure of a certain bacterial strain to a single phage is suggested to support phage resistance.	[18]	Task for applied research. Evaluation needed. Use of phage cocktails.
Negative influence of CRISP-Cas9 action on phage activity (*e.g*. in *L. monocytogenes*)	[16]	Observation under experimental conditions. Not relevant on foodstuff (?)
How long are the phages stable/active?	[13]	Mostly, phages are not intended to “survive.” Phages lyse bacteria just after application for a certain time. Due to immobile phages after application, newly emerging bacteria might not be reached on solid food.
Prolonged phage stability/activity? Can long-lasting protection against recontamination or regrowth be ensured	[4], [15]	The conditions enabling prolonged phage stability/activity must be clarified. Prolonged stability/activity must be proven. Task for applied research. Evaluation needed.
Possible difficulties to detect pathogens in phage-treated samples.	[13], [14]	QPS systems for culture-based detection and registration of phage use might be needed.
Phage “survival” in the food chain as well as within the cold chain. Fate of phages in the environment after application in livestock and food facilities. Risk of high concentrations of individual phages and coevolution of bacteria and phages in the environment, shift in microbial community composition?	[3], [13]	Pathogens and spoilage bacteria are targets of phage use, killing is desirable. The target species only make up a small subset of the population; therefore, no change is expected. Research need.
Unknown genes of phages.	[5], [12]	Genome of used phages should be devoid of genes coding for virulence factors and antibiotic resistance. Sequencing of phage genome. Responsibility of companies according to good manufacturing practice (GMP).
***Possible risks for health***		
Influence of chronic intake of phages on the gut microbiota.	[2]	Not yet observed. Further research needed.
Is the dissemination of phages and their interactions occurring within the human body, e.g. with the intestinal mucus layers, a risk? Can coevolution of bacteria and phages in the intestine change the intestinal microbiota?	[13]	Not yet described. Phages are daily taken up via food. Phages for use on food kill undesirable pathogens, not naturally occurring in the intestine. Research needed.
Release of pro-inflammatory compounds and toxins from lysed bacteria.	[2], [4]	Not yet observed. Further research needed.
Allergenicity	[6], [7], [12], [16]	Experimental results that rule out allergenicity of phage proteins. Not yet observed. Not likely via oral administration.
***Further concerns***		
Consumer acceptance	[12], [1], [7]	Knowledge must be comprehensive for consumer education. Marketing problem.

## Properties of phages relevant for their application in the food sector

Phages used for therapy or to reduce bacterial contamination in the food production chain belonged to the formerly valid order *Caudovirales*, the largest group of known phages (≥ 90%), which were subdivided into the families “*Myoviridae*,” “*Podoviridae*,” and “*Siphoviridae*” (Sharp 2001). All tailed phages are now assigned to a single class (*Caudoviricetes*), which comprises a large number of families and subfamilies (Turner et al. 2021). Some properties relevant to their use in the food sector (Hagens and Loessner 2010, Wang and Zhao 2022) are outlined below.

Phages infect bacteria or archaea and are characterised by their generally **high host specificity** (Ackermann 2001, Koskella and Meaden 2013, Taslem Mourosi et al. 2022). This specificity can be very narrow, i.e. limited to certain strains or serotypes of a species, or it can be broad, encompassing different species or even genera. The receptor binding proteins (RBP) bind specifically to receptor molecules on the surface of bacteria according to the key/lock principle (Stone et al. 2019). Among other classifications, phages can be divided into two groups, depending on their life cycle, namely virulent and temperate phages (Hobbs and Abedon 2016). Only **virulent phages with strictly lytic reproduction** are generally chosen for use in the food sector. They consistently follow the same reproduction cycle in the bacterial cell, which is subsequently lysed to release newly synthesized virions.

Regardless of whether they are temperate or virulent, phages can incorporate bacterial DNA into their capsids and transfer it to other bacteria due to errors in packaging of newly synthesised phage genomes at the end of the lytic cycle (generalized transduction). Although rare, **random packaging of bacterial DNA** into the capsid of virulent phages and its dispersal may occur. Due to their close contact with the bacterial chromosome, temperate phages are more capable of transduction (specialized transduction) than virulent phages (Brabban et al. 2005). This has led to some temperate phages permanently carrying genes of bacterial origin (e.g. virulence or antibiotic resistance genes) in their genome. If such phages enter the lysogenic cycle, the new host can express these genes and may therefore change its phenotype (lysogenic conversion) (Brüssow et al. 2004). Because of this capacity for targeted transduction, temperate phages are generally not suitable for safe applications in food production (Cadamuro et al. 2025).

Just as the host range of individual phages can differ from one another, so can their lytic activity. The efficiency and rate of **phage-mediated lysis** depend on several factors, for example on the time required for binding to the cell, which is influenced by the affinity and accessibility to the receptor. Other important factors include the time between adsorption to the host cell and the release of newly formed phages (latent period), the number of phage particles released per cell (burst size) and the activity of lytic enzymes (endolysins) synthesised by phages at the end of infection. In addition, the infected host cell can strongly influence the lytic activity, e.g. through slow growth, altered receptors, or phage defence mechanisms such as CRISP-Cas (Díaz-Muñoz and Koskella 2014).

Two types of killing are described: (1) lysis after phage multiplication in the cell (“**lysis from within**”) (Young 2014); and (2) damage of the cell wall due to the specific binding of excessive phages to the bacterial surface (“**lysis from without**”) (Rakhuba et al. 2010). The latter occurs solely by damage of the bacterial cell wall. Not all phages are capable of killing bacteria by this process (Abedon 2011). In applications on food surfaces, the number of phages required to achieve “lysis from without” is assumed to be difficult to reach (Fischer 2019, Lewis and Hill 2020). In contrast, “lysis from within” requires multiplication in a metabolically active bacterial cell (Bryan et al. 2016). Another important property of phages is their ability to coevolve with changing bacterial populations (Borin et al. 2021, Borin et al. 2023). This includes the **coevolution of RBPs** in response to emerging phage resistance of the host bacteria. As a result, phages with an appropriate host range can consistently be isolated from the environment when new or modified strains or serotypes of a bacterial species appear.

Phages persist both inside and outside the bacterial cell (Batinovic et al. 2019). The **stability of phages** outside the cell, where they exist as inanimate, but still infectious particles, is crucial for successful application. The chemical and structural properties of the capsid and the tail determine the stability of the phage particles to changes in pH, salinity, temperature, and desiccation.

An annotated glossary of key terms and definitions relevant to phage applications is available (Abedon 2025).

## Phage application in the food sector

**Figure 1.**
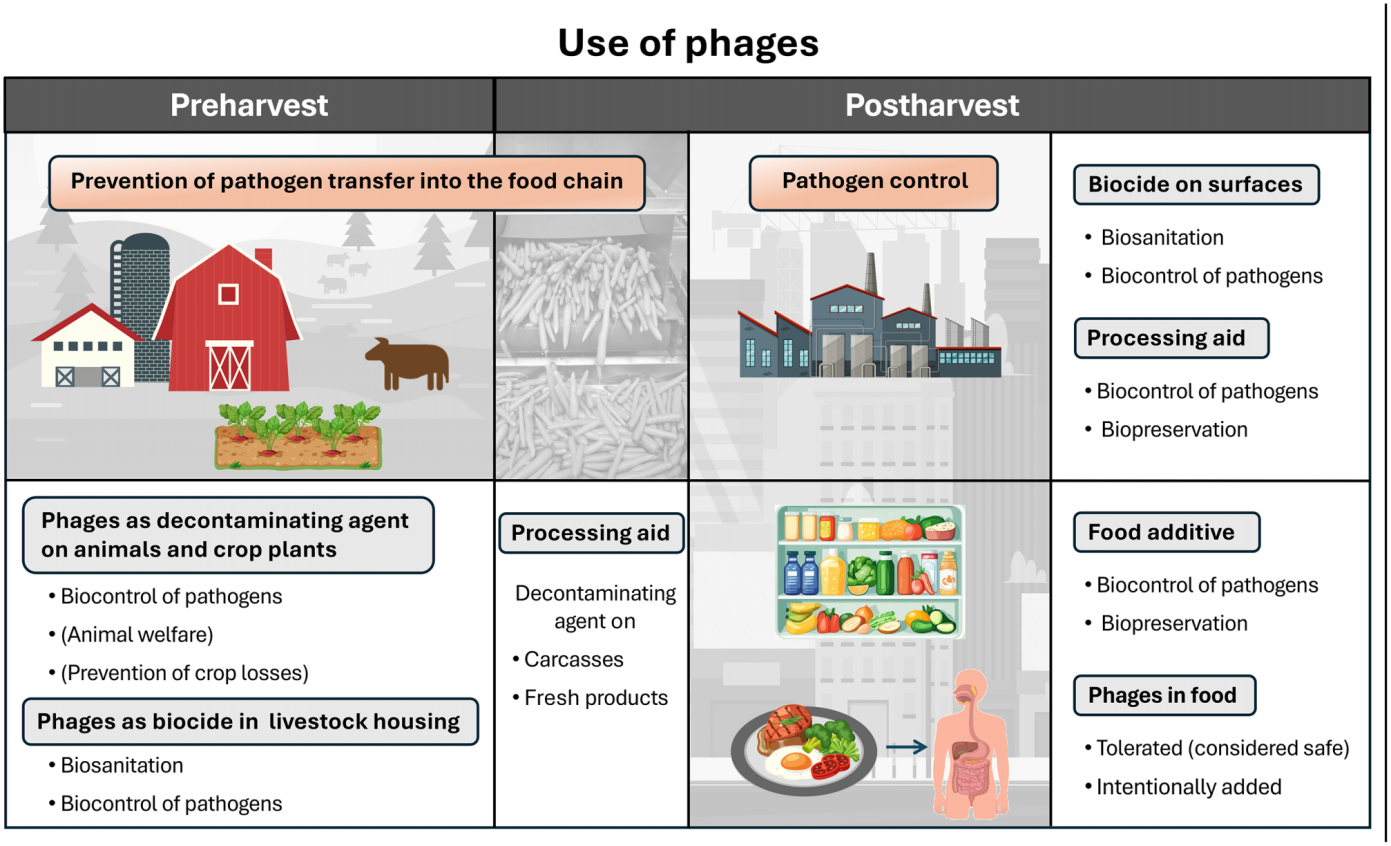
Different stages of food production for potential phage applications. Left: Phage applications in the preharvest sector (animal husbandry and crop cultivation). Middle: Phage applications during slaughter and harvest (transitional stage from pre- to postharvest). Right: Phage applications in the postharvest sector (processing and finished products). Bottom right: Phages in food, either remaining and tolerated after their use as a processing aid or intentionally added as a food additive.

In principle, phages can be applied at various stages of food production, including treatment of the final product within its packaging (Cooper 2016, Mills et al. 2017, Moye et al. 2018, Gutiérrez et al. 2019, Cristobal-Cueto et al. 2021, Gildea et al. 2022). A distinction is made between applications in crop cultivation and animal husbandry, commonly referred to as ‘preharvest’ in the context of food safety (Torrence 2016), and applications within the value chain or food production chain, referred to as “postharvest” (Fig. 1) (Greer 2005, Hagens and Loessner 2010, Goodridge and Bisha 2011, Endersen et al. 2014, Cristobal-Cueto et al. 2021, Gildea et al. 2022, Jagannathan et al. 2022). References summarizing phage applications in the food sector are listed in the Supplementary Table S1. Depending on their intended purpose, phage applications can be classified as biocontrol, biopreservation, or biosanitation, each of which may be conducted either pre- or postharvest (García et al. 2008, Kazi and Annapure 2016).

## Biocontrol, biopreservation, and biosanitation

**Biocontrol** by phages refers to the targeted reduction of foodborne pathogenic bacteria on food and production surfaces. It represents the most important objective of phage application in the pre- and postharvest sector. In the preharvest sector, applications in animal and crop production aim to prevent pathogen transmission into the food chain (Fig. 1) (Goodridge and Bisha 2011, Sulakvelidze 2013, Endersen et al. 2014, Moye et al. 2018, Vikram et al. 2021, Bumunang et al. 2023, Imran et al. 2023, Amjad et al. 2024, Chaudhary et al. 2024). The term *biocontrol* is also used in the context of animal and plant therapy (de Melo et al. 2018). Some authors also include the reduction of food spoilage bacteria under the term *biocontrol* (Cristobal-Cueto et al. 2021), although this usage extends beyond the original definition focused on human pathogens.

**Biopreservation** (also rarely referred to as bioconservation) by phages is the targeted reduction of microorganisms that cause food spoilage, which is reflected in changes in the food matrix and taste (Pérez Pulido et al. 2016). Microbiological food spoilage is a multifactorial process driven by metabolically diverse microbial communities, which fundamentally complicates the use of phages for spoilage prevention. Nevertheless, phages can complement other preservation strategies within a hurdle concept (García-Anaya et al. 2020, Moye et al. 2018). To date, only certain critical or dominant spoilage organisms have been the focus of phage-based biopreservation. Phages can be useful when a single bacterial species constitutes the dominant spoilage microbiota under specific ecological conditions, such as *Pseudomonas* spp. in raw milk (Hu et al. 2016, Pérez Pulido et al. 2016) or *Brochothrix thermosphacta* in pork (Greer and Dilts 2002, Pérez Pulido et al. 2016). Occasionally, the term biopreservation is used less precisely to also refer to the reduction of human pathogens. Human pathogens generally do not alter food properties and therefore do not contribute to food spoilage (NRC 1985, Snyder et al. 2024).

**Biosanitation** by phages is the decontamination of microbially contaminated surfaces in animal housings, as well as work surfaces and equipment made of plastic and steel that may contribute to food contamination during production (Sevilla-Navarro et al. 2020, Reinhard et al. 2023). Of particular interest is the reduction of biofilms, which are a major cause of recontamination in food-processing environments (Nannapaneni and Soni 2015, de Ornellas et al. 2017, Gong and Jiang 2017, Sadekuzzaman et al. 2018, Cha et al. 2019, Gutiérrez et al. 2019, Islam et al. 2019, Garvey 2022, Gildea et al. 2022, Liu et al. 2023). Misleadingly, the targeted reduction of bacterial contamination by phages on surfaces or in water is sometimes referred to as bioremediation (Cristobal-Cueto et al. 2021). Bioremediation, however, is the biological removal of pollutants, *e.g*. by microorganisms that metabolize them or by isolated phage tail protein capable of binding metal ions (Shahwar et al. 2024). References addressing biocontrol, biopreservation and biosanitation by phage applications are listed in the Supplementary Table S1.

## Preharvest phage applications in livestock and crop cultivation

In animals, on surfaces of animal housing, or on leaf surfaces of crop plants, phage applications can reduce contamination of human pathogenic bacteria and thereby decrease their transmission into the food value chain, improving food safety (Fig. 1) (Hagens and Loessner 2010, Huang and Nitin 2020, Lu et al. 2020, Gildea et al. 2022, Bumunang et al. 2023). Phages can also be used therapeutically in farm animals or crops (El-Shibiny and El-Sahhar 2017, Gutiérrez et al. 2019, Cristobal-Cueto et al. 2021) for conditions such as bovine mastitis, rhinosinusitis in sheep, *E. coli* O157:H7 infections in ruminants, or salmonellosis and colibacillosis in chickens (Vander Elst and Meyer 2018, Fong et al. 2019, Harshitha et al. 2022). On plants, phages can be applied to control diseases such as citrus and tomato canker or apple and peach tree blight (Holtappels et al. 2019, Garvey 2022) and in aquaculture against *e.g. Citrobacter freundii, Aeromonas hydrophila*, or *Vibrio* spp. (Jaglan et al. 2022, Lavilla et al. 2023, Lomelí-Ortega et al. 2023), to prevent production losses. However, phage therapy is not the focus of this article. References addressing phage applications in the preharvest sector are listed in the Supplementary Table S1.

## Postharvest phage applications in the food production chain

The postharvest sector begins with the preparation of raw materials and includes food processing up to the finished product (Fig. 1) (García et al. 2008, Goodridge and Bisha 2011). Phage applications can be carried out on the raw material or intermediate product as an additional treatment in combination with other measures (Bumunang et al. 2023; García-Anaya et al. 2020; Tan et al. 2014, Shymialevich et al. 2024). Phages can also be applied to finished food products prior to packaging or incorporated into the packaging material itself (O'Sullivan et al. 2019, Ahmadi et al. 2020, Wang and Zhao 2022, Wagh et al. 2023). In contrast to applications within the production line, where removal of phages may be possible and is sometimes discussed (García-Anaya et al. 2020), the removal or inactivation of phages on finished food products, such as sliced meat, cheese, fruits and vegetables, beverages, and RTE products, is not intended, as the food properties and/or phage activity are to be preserved. Consequently, consumers ingest phages. References addressing phage applications in the postharvest sector are listed in the Supplementary Table S1.

## Relevant bacterial species in the food sector

For nearly all pathogenic or spoilage-causing bacterial species of the food sector, phages can be isolated (Greer 2005, Pérez Pulido et al. 2016, de Melo et al. 2018, O'Sullivan et al. 2019, Lewis and Hill 2020, Cristobal-Cueto et al. 2021, Ramos-Vivas et al. 2021, Ge et al. 2022, Liu et al. 2023). Significant human-pathogenic bacteria on food include the three most important representatives of the *Enterobacterales, Salmonella enterica*, toxin-producing *E. coli*, and *Yersinia enterocolitica* but also *Shigella* spp., as well as *L. monocytogenes, Campylobacter* spp., and *S. aureus* (EFSA 2022). In 2024, EFSA identified *L. monocytogenes* (in the meat, fish, seafood, dairy, fruit, and vegetable sectors), *S. enterica* (in the feed, meat, egg and low-moisture food sectors), and *Cronobacter sakazakii* (in the low-moisture food sector) as the bacterial food safety hazards most relevant to public health, associated with persistence in the food and feed processing environment (EFSA 2024).

For a long time, it was difficult to develop phage preparations against *Campylobacter jejuni* for practical application. Such phages lysed *Campylobacter* only insufficiently, which was attributed to the high diversity of *C. jejuni* subpopulations (Ushanov et al. 2020), in particular capsule variants with altered sensitivity to capsule-specific phages. Additionally, *Campylobacter* spp. are both microaerophilic and thermophilic, making them difficult to cultivate, especially in large quantities for phage production (Hofreuter 2014, Jäckel et al. 2019, Ushanov et al. 2020). Due to their environmental requirements, *Campylobacter* cells are often not metabolically active on food and therefore not susceptible to phage infection, which complicates phage-mediated killing (Tabashsum et al. 2024).

No phage cocktails are currently approved for use against *Y. enterocolitica*, a species found predominantly in pork and responsible for intestinal infections. However, some phage isolates that lyse this species have been tested on various foods (Orquera et al. 2012, Jun et al. 2018, Leon-Velarde et al. 2019).

*Staphylococcus aureus* and *B. cereus* can spoil food and produce toxins leading to food poisoning. Both can form biofilms on surfaces. Promising phage isolates have been developed against *S. aureus* (Sulakvelidze 2013, Cha et al. 2019, Duc et al. 2020), while only a few phages are known for *B. cereus*, which occurs ubiquitously in soil and dust and produces enterotoxins when it multiplies in food or the intestine. Some studies have investigated the efficacy of phages against *B. cereus* on foodstuffs (Bandara et al. 2012, Nakonieczna et al. 2015, Hock 2019, Artawinata et al. 2023) and in biofilms on surfaces (Gdoura-Ben Amor et al. 2022).

Phage isolates have also been described that are effective against *Cronobacter sakazakii* (Endersen et al. 2017, Vikram et al. 2021), a contaminant in milk and milk powder. Recently, specific phages for enterococci have been reported (Wandro et al. 2022). While some enterococci are beneficial for food fermentation, others may pose health concerns due to the production of toxic biogenic amines in food and in the gut (Rodríguez-Lucas and Ladero 2023). Other bacterial species suitable for the use of phages in the preharvest sector are *Clostridium perfringen*s, which occurs in animal housing and contaminates fresh meat, and *Vibrio parahaemolyticus*, which contaminates fish and seafood (Lee et al. 2023). References providing an overview of pathogens and spoilage bacteria, and the appropriate phages and phage cocktails are listed in the Supplementary Table S1.

## Isolation, screening, and mass production of phages

Phages included in phage cocktails are mostly isolated from the environment, particularly from animals or food. Phages exhibiting the broadest host range and high lytic activity are screened for and selected. Other important properties of phages include tolerance to a wide pH range, thermal stability, and a high level of reproductive activity with a short “latent period” and a large “burst size” (ideally around 100 phages per cell) (Islam et al. 2019). The basic requirement for mass production is that phages can be propagated with satisfactory efficiency on host strains.

For the industrial production of phages, the cultivation conditions established by scientists for small-scale cultures in the millilitre range must be scaled up (García et al. 2019, Wiebe et al. 2024). For obvious reasons, no data on phage production are available from the industrial sector. According to the available information, bacterial cultures with a volume of 100 L (depending on the species ∼ 10^7^ to 10^8^ bacterial cells mL^–1^) are infected with 1 L of phage suspension at the beginning of the logarithmic growth phase (personal communication with a manufacturer). To achieve the average required MOI (multiplicity of infection: phage particles per target cell) of 1–5, the phage suspension must contain 10^9^ to 10^10^ phage particles mL^–1^. Nevertheless, the optimal MOI of inoculation may vary depending on factors such as the phage species. Scaling up a phage culture in three steps from 100 µl to 1 L to infect 100 L cultures is also possible. Depending on the burst size, phage enrichments with more than 10^10^ phage particles mL^–1^ can be achieved (García et al. 2019). Phages are purified by successive filtration and centrifugation steps, which separate intact bacteria and, depending on their size, membrane fragments. Further purification steps include precipitation (e.g. with polyethylene glycol), ultracentrifugation, dialysis, and affinity chromatography. These methods have the potential to remove macromolecules such as endo- and exotoxins as well as low-molecular-weight components, although some of them might be challenging to remove from large-scale production (Branston et al. 2012, Bourdin et al. 2014, Hietala et al. 2019, Fouladvand et al. 2020).

## Phage modification, *in vitro* evolution, and genetic engineering

Besides screening for naturally occurring phages, various strategies are being investigated to obtain phages with enhanced properties (reviewed by (Lee et al. 2023). In the method known as “*in vitro* evolution,” recombined phages with newly combined favorable properties can be produced by co-infecting bacterial suspensions with different phages (Poullain et al. 2008). Phages with enhanced properties for the reduction of phage-resistant *L. monocytogenes* were obtained by co-infection of *L. monocytogenes* suspensions with phages of an orthocluster. The average sequence homology of this orthocluster (approximately 91%) facilitates recombination (Peters et al. 2020). There are studies, in which isolated phages have been genetically modified to enhance their application properties (Dunne et al. 2019). However, the commercial use of such genetically engineered phages is not allowed in the EU (Regulation 1829/2003 on genetically modified food and feed) and has not yet been approved in the USA or other non-EU countries. Research is also ongoing to re-engineer phages for specific requirements, producing phages that only possess genes essential for host cell recognition, infection and lysis, and phage assembly and propagation (Lenneman et al. 2021).

## Phage cocktails

Phages and phage cocktails targeting foodborne pathogenic bacteria, and to a lesser extent spoilage bacteria are currently either (i) with Generally Recognized As Safe (GRAS) status, some of them approved as processing aids in the USA and several countries outside the EU, and available there as single phage preparations or phage cocktails (Table 2); (ii) positively tested on a variety of foods but not yet with GRAS status or approved for use in any country (Hyla et al. 2022, Bumunang et al. 2023); or (iii), so far, only isolated and scientifically characterised, including assessments of their host specificity and killing efficiency against food relevant bacterial species (Pereira et al. 2017, Martinez-Soto et al. 2024). Most approved phage cocktails are directed against the genera *Salmonella, Escherichia*, or *Listeria* (Table 2). Due to the large number of different strains and serotypes of the species (Croxen et al. 2013, Tanner and Kingsley 2018), and the limited host range of individual phages, commercial phage cocktails against these pathogens typically consist of two to six different phages (Endersen and Coffey 2020). The number of different phages with distinct host specificities that can be included in a phage cocktail is limited by the maximum concentration that should be achieved for each phage in the final formulation and by the maximum volume that can be applied. A high titre must be maintained for each individual phage; consequently, the inclusion of additional phage inevitably dilutes the others. For *L. monocytogenes*, two phage products are available (Table 2). One of them (Phageguard L^TM^) consists of a single phage, whereas the other (Listshield^TM^) contains six phages. Since 2021, a phage cocktail consisting of three to eight phages active against *C. jejuni* and *C. coli*, further important food associated pathogens and the primary causes of foodborne illness from chicken meat in Europe, has been GRAS-designated by the Food and Drug Administration (FDA) and is intended for application on raw meat and poultry (Table 2).

**Table 2.** State-by-State overview of regulatory approval, classification, and information on phage cocktails.

State	Phagecocktail/Company	GRAS status/approved	Terms of use
USA by FDA or USDA	***Listeria monocytogenes***		
	ListShield™/ Intralytix	**GRAS-status** * by FDA (GRN 528) **Food additive** under 21 CFR § 172.785 by FDA **Processing aid** with FSIS Directive 7120.1	Ham, sausages, poultry products, fish and seafood fresh and processed fruits and vegetables
	PhageGuard L™ (Listex P 100)/ Micreos	**GRAS status** * by FDA (GRN 198, GRN 218 for supplemented cocktail)	Meat and poultry products, cheese (e.g. on the rind of hard cheeses), fish and seafood fresh and processed fruits and vegetables
	***Escherichia coli* O157:H7**		
	PhageGuard E™/ Micreos	**GRAS status** * by FDA (GRN 757) **Processing aid** with FSIS Directive 7120.1	Beef carcasses, meat-trimmings, vegetables
	EcoShield™/Intralytix	**GRAS status** * by FDA (GRN 834)	Red meat parts, trim for ground, as meat, poultry, dairy products fruits and vegetables
	***Salmonella enterica***		
	PhageGuard S™ (Salmonelex)/Micreos	**GRAS status** * by FDA (GRN 468, GRN 859 for supplemented cocktail) **Processing aid** with FSIS Directive 7120.1	Certain pork and poultry products; beef vegetables fresh and saltwater seafood
	SalmoFresh™ (former SalmoShield)/Intralytix	**GRAS status** * by FDA (GRN 435) **Processing aid** with FSIS Directive 7120.1	Reduction of *Salmonella* on food
	SalmoPro™/Phagelux	**GRAS status** * by FDA (GRN 603, GRN 752 for supplemented cocktail)	Poultry products, on poultry surfaces (max 10^8^ PFU/g)
	PhageFend™/Cytophage	**GRAS status** * by FDA (GRN 1163)	Raw poultry before processing
	Applied Phage Meat S2/Fink Tec GmbH	**GRAS status** * by FDA( GRN 1038)	Raw meat, poultry, carcasses, primals, subprimals, trimmings (10^5^–10^7^ PFU/g)
	Applied Phage Vegetable S2/Fink Tec GmbH	**GRAS status** * by FDA (GRN 1070)	Fresh and processed fruits and vegetables
	*S*. Enteritidis Phage Prep./Qingdao Phagepharm Bio-Tech Co	**GRAS status** * by FDA (GRN 1134)	Ground chicken
	***Shigella* spp**		
	ShigaShield™/Intralytix	**GRAS status** * by FDA (GRN 672)	On foods susceptible to Shigella contamination as fresh produce, meat, and poultry
	***Campylobacter jejuni, C. coli***		
	Campyshield ™/Intralytix	**GRAS status** * by FDA (GRN 966)	Red meat: whole carcasses, primal cuts, trimmings, organs; raw poultry: carcasses and parts (max 10^8^ PFU/g)
Canada by Health Canada	***Listeria monocytogenes***		
	PhageGuard L™/Micreos	**Processing aid** by LONO (GRN 198)	Surface treatment of meat, fish, cheese, RTE products
	ListShield/Intralytix	**Processing aid** by iLONO (GRN 528)	Meat, fish, poultry, plant-based RTE products
	***Escherichia coli* O157:H7**		
	EcoShield™/Intralytix	**Processing aid** by iLONO (GRN 834)	Red meat parts and trim prior to grinding
	***Salmonella***		
	PhageGuard S™/Micreos	**Processing aid** by LONO (GRN 468)	Poultry, meat, fish, fruit/vegetables
	SalmoFresh™/Intralytix	**Processing aid** by LONO (GRN 435)	
	PhageFend™/Cytophage	**Processing aid** by FDA (GRN 1163)	Raw poultry before processing
	OvaPhage™/Cytophage	**Processing aid** by LONO (no GRN)	Surface treatment of eggs
Israel	***Listeria monocytogenes***		
	PhageGuard L™/Micreos	**Processing aid** (GRN 198)	Decontamination on food
	ListShield/Intralytix	**Processing aid** (GRN 528)	Decontamination on food
	***Escherichia coli* O157:H7**		
	EcoShield™/Intralytix	**Processing aid** (GRN 834)	Treatment of meat immediately before grinding
	***Salmonella***		
	PhageGard S™/Micreos	**Processing aid** (GRN 468)	Decontamination on food
	SalmoFresh™/Intralytix	**Processing aid** (GRN 435)	Decontamination on food
Switzerland	***Listeria monocytogenes***		
	PhageGuard L™/Micreos	**Processing aid** (GRN 198)	Cheese production
Netherlands	***Listeria monocytogenes***		
	PhageGuard L™/Micreos	**Processing aid** (GRN 198)	Raw meat and poultry, cheese production
Australia/New Zealand by FSANZ	***Listeria monocytogenes***		
	PhageGard L™/Micreos	**Processing aid** (GRN 198)	RTE-products
	***Salmonella***		
	PhageGard S ™/Micreos	(GRN 468)/**processing aid**	Raw meat and raw poultry meat
India	***Salmonella***		
	PhageGard S™/Micreos	**Processing aid** (GRN 468)	For food (not specified,) on food contact surfaces
Egypt	PhageGard S™/Micreos	**Processing aid** (GRN 468)	Poultry production
USA (Preharvest)	***Escherichia coli* STEC**		
	EcoShield PX™/Intralytix	GRN 834 extended/n.k.	Animal (“Preharvest), partially also on food
	PhageGuard E Hides/Micreos	**Processing aid** by FSIS Directive 7120.1	On animals (“Preharvest”)
EU, Brazil (Preharvest)	***Salmonella***		
	Bafasal/Proteon	**Feed additive**	Poultry feed (Preharvest)

* Generally Recognized As Safe for human consumption; EU: European Union; (US-)FDA: (US-)Food and Drug Administration; FCN: Food Contact Notification; FSANZ: Food Standards Australia New Zealand; FSIS: Food Safety and Inspection Service of USDA; GRAS: Generally Recognized As Safe; GRN: GRAS Notice Number; (i)LONO: (interim)Letter of No Objection; n.k.: not known; STEC: Shiga toxin-producing *E. coli*; USDA: United States Department of Agriculture. (References: official sites of authorities; manufacturer information).

Further phage preparations are either already available or under development against species belonging to the genera *Streptococcus, Staphylococcus, Cronobacter, Xanthomonas, Pseudomonas, Aeromonas, Agrobacterium, Dickeya, Xylella, Pectobacterium, Erwinia*, and *Clavibacter*, which contribute either to food spoilage or to plant diseases (Holtappels et al. 2019, Połaska and Sokołowska 2019, Xu 2021).

Because phage resistance may emerge, or other strains and serotypes may become predominant (a phenomenon particularly common in *Enterobacteriaceae*), new phage isolates are continually tested as potential replacements in already approved phage cocktails. This ensures that efficacy is maintained and that the spectrum of activity can be expanded (Zhang et al. 2023).

## Conditions for phage applications on foodstuffs

The efficiency of phages depends not only on the phages and target bacteria, but also on the food matrix and variant properties, such as the state or consistency of the food (i.e. liquid or solid) or its surface (i.e. smooth, furrowed) (Guenther et al. 2012, Kawacka et al. 2020). Surfaces with heterogeneous structures, where bacteria are unevenly distributed and only partially accessible, are included in modeling systems to examine the probability of phages encountering their target cells. In these models of spatially structured surfaces, “cells-first” scenarios are typically used, as is usually the case in the application of phages to contaminated foods. Smooth, easily wettable surfaces promote good mixing of phages and bacteria, enabling a more complete reduction of the host cells. However, when bacteria reside in microscopic niches, such as furrows, cracks, pores or films of fat or water found on food, phages that do not infect their host, and either remain free or adsorb non-specifically to the surface (Guenther et al. 2009) may be lost over time, while some of the bacteria survive. Under certain spatial conditions, however, an equilibrium can develop in which phages and bacteria coexist. In such cases, both phages and bacteria persist, and bacterial numbers are not significantly reduced (Eriksen et al. 2019, Fischer 2019, Sinha et al. 2020, Hunter et al. 2021, Valdez et al. 2025). Excess phages at high titres are predicted to eliminate host cell populations rather than allow coexistence (Eriksen et al. 2019, Fischer 2019). Due to these uncertainties, high standard concentrations are recommended for phage suspensions applied to food surfaces in order to increase the likelihood of encounters between phages and their hosts (Fischer 2019, Lewis and Hill 2020). Techniques such as dipping, spraying, or moistening the food have been described to achieve a more uniform distribution of phages during application (Sulakvelidze 2013, Kazi and Annapure 2016, Pérez Pulido et al. 2016, Moye et al. 2018, Lewis and Hill 2020, Pinto et al. 2020, Cristobal-Cueto et al. 2021, Garvey 2022, Lavilla et al. 2023).

The temperature, pH and salt content are additional factors that can influence effectiveness (Liu et al. 2023, Ranveer et al. 2024). Manufacturers recommend specific temperature ranges for application. For example, EcoShieldPX^TM^ is recommended for use between 2°C and 42°C. Phages are not effective under extreme cooling, as the host cell, depending on the species, must be metabolically active (Bryan et al. 2016).

A distinction must be made between the stability and the actual activity after application to the food. Stability, defined as the ability to infect and lyse cells, can persist over extended periods. For instance, phage P100 (used as PhageGuard Listex^TM^) has been reported to remain stable in smear water, at temperatures ranging from 4°C to 10°C for up to 120 days (Fister et al. 2016). According to studies, the duration of phage activity, referring to the infection and lysis of host cells, varies after application depending on the phage cocktail, the metabolic activity of the host, and the food environment. Some authors describe phages as active only within a limited period, from several hours up to 48 hours after application without preventing bacterial regrowth (Kawacka et al. 2020, Amjad et al. 2024). Others report activity lasting more than 48 hours, including the ability to reduce regrowth or subsequent contamination with the target bacteria (Yang et al. 2017, Ishaq et al. 2020, Zhou et al. 2020, Colás-Medà et al. 2023).

In certain applications such as liquid foods or foods floating in liquids, phages may undergo multiple infection cycles, infecting and killing resurgent, actively growing host cells, whereas on solid foods, phage multiplication typically occurs in a single infection cycle in the hosts reached and infected immediately after application. Due to the low bacterial counts and their uneven distribution on solid foods, a negligible multiplication of phages is expected (Hagens and Loessner 2010) and phages released from lysed hosts account for only about 1% of the initially applied phage quantity (Guenther et al. 2012).

Contaminations are expected to occur sporadically and unevenly across the surface, making contact with already applied, still-active phages unlikely, as phages generally do not actively move toward target cells. Even if such effects may occur under certain, potentially unpredictable conditions, phage cocktails should not be applied with the aim of combating recurring bacterial growth or new contamination after their application (“phage-first” scenario). The potential for continuous lysis over extended periods after application, particularly in cases of regrowth or subsequent contamination, remains uncertain (Hagens and Loessner 2010, Kawacka et al. 2020).

In liquid foods, phage immobilisation may depend on the quantity and size of suspended solids. The addition of phages to liquids, such as mozzarella in its brine during storage, appears to be a promising application. References providing an overview of inoculated food items are listed in the Supplementary Table S1.

Phage treatment cannot and must not be used to render food marketable again once it is already heavily contaminated with bacteria. Although several log-unit reductions can be achieved under conditions of high bacterial loads, the outcome remains too inconsistent to reliable ensure the microbiological safety of the food product (Moye et al. 2018).

Phage application can also be combined with bacteriocins (e.g. pediocin, nisin) or bacteriocin-producing bacteria, as well as with complex tannins or *trans*-cinnamaldehyde (Viazis et al. 2011, Heo et al. 2018, Kim et al. 2019, Bumunang et al. 2020, Duc et al. 2020, Rendueles et al. 2022). Methods for immobilising phages on packaging material, or on pads placed within the packaging alongside the product have been described (Gouvêa et al. 2016, Lone et al. 2016, Wagh et al. 2023), as well as embedding of phages in coatings of alginate or xanthan gum (García-Anaya et al. 2023). When phages are added to the packaging or embedded in the packaging material, the aim is to maintain their activity over prolonged storage. Further approaches to stabilise phages and thereby improve their delivery and maintain their activity are currently under investigation (Xu 2021, Costa et al. 2023, Liu et al. 2023).

## Phage/host ratio

Besides the conditions of the food environment and the diversity of the potentially present strains and serotypes of a bacterial species, the efficiency of phage application is crucially influenced by the phage/cell ratio. Cell lysis requires that at least one phage reaches and absorbs to the host cell and completes one round of multiplication. MOI values as defined for bacterial suspensions, cannot be directly transferred to surface applications (Abedon 2016). The actual number of phages that reach a given cell is much lower on solid surfaces and is determined largely by factors such as the spatial distribution of the bacteria (whether they occur individually or in clusters), their local density, surface properties, and diffusion processes. To obtain more realistic predictions, biomechanical model systems can be used to simulate when, where, and to what extent phages encounter their host cells, particularly within bacterial colonies and biofilms (Valdez et al. 2025).

Many studies using solid-food models report MOI values calculated from the sequentially applied amounts of bacteria (CFU) and phages (PFU), even though these MOI values have limited interpretive value. Such calculation implicitly assumes that phages encounter host cells as uniformly as in liquid cultures at appropriate phage and bacterial concentrations, where both are well mixed, an assumption that is not valid for solid surfaces even when food is inoculated. As in liquid systems with low host-cell densities (Bigwood et al. 2009), a substantial surplus of phages is required, rendering the MOI largely irrelevant (Fischer 2019).

Reported MOIs in such studies with solid-food models ranged from 1 to 10^6^, with occasional values as high as 10^8^ (Jayamanne and Foddai 2025). Although reductions of 1–4 log₁₀ in surface-recovered target bacteria generally increase with these calculated MOIs, this relationship may be misleading. The observed effect likely results from the greater surplus of phages inherently associated with higher MOI calculations rather than from the MOI concept itself. It should be noted that the MOIs and the reduction rates reported for solid-food model systems are hard to compare. This is not only due to differences in the food models themselves, but also to variations in how bacterial and phage suspensions are applied (e.g. by dipping inoculated food into phage suspensions or by spraying,) and because concentrations are expressed in different reference units (CFU or PFU per cm^2^ or g^1^ of food). Phages at high concentrations, such as when “lysis from without” is intended, can clump together under certain conditions, for example at low salt concentrations. This aggregation may cause a temporary and reversible loss of infectivity (Szermer-Olearnik et al. 2017). When bacterial cells form clumps, the effects can vary. If these clumps consist exclusively of target host cells (clonal bacterial clumps), phage activity may actually increase, as the high local density of metabolically active cells facilitates successive lytic cycles (Abedon 2017). In contrast, mixed species cell clusters may impede phage efficacy. When host cells are surrounded or shielded by non-host bacteria, phages may fail to achieve the required local host-cell density needed to initiate productive infection, thereby reducing or preventing lytic activity. References providing information on the effectiveness of phages and phage cocktails are listed in the Supplementary Table S1.

## Sustainability of phage application

Finally, the question arises whether the use of phages can enhance the sustainability of food production in addition to improving microbiological safety. On the one hand, phage-based interventions require energy and resources, for example, for phage propagation, and involve production costs such as safety testing, storage, and packaging (Torres-Acosta et al. 2019). On the other hand, comparisons of different food-processing technologies indicate that phage treatments can be more efficient in terms of energy and resource use than several conventional measures. For example, the low-impact decontamination methods such as irradiation or high-pressure processing, cost between 0.22 and 0.66€ per kg of food. In contrast, phage-based decontamination is estimated to cost only 0.02 to 0.09 cents per kg, placing it in a range similar to that of more potent disinfectants (Vikram et al. 2021). Cost assessments have also been carried out for the development and large-scale production of phage preparations (Krysiak-Baltyn et al. 2018), including applications as controlling *Salmonella* in poultry farming (Torres-Acosta et al. 2019). Phages may further contribute to resource efficiency by reducing food waste, for example, by lowering contamination levels of milk, dairy products, carcasses, and raw meat. Their use for biofilm control in production environments, such as in tanks and containers, has also been described as more sustainable than repeated disinfection and rinsing steps. In the context of phage therapy in the livestock sector, although not the subject of this statement, the potential reduction in antibiotic use is considered both beneficial and sustainable (Gutiérrez et al. 2019). Likewise, the reducing crop losses due to spoilage or plant disease through phage application is discussed as an additional sustainability benefit (Alvarez and Biosca 2017). A reduction in medical treatment costs for food-borne diseases may also be a beneficial consequence of phage use. For instance, lowering *Campylobacter* contamination on broiler carcasses by 2 log units is expected to significantly reduce the incidence of *Campylobacter* infections (Rosenquist et al. 2013).

## Safety aspects related to the use of phages in food production

**Figure 2.**
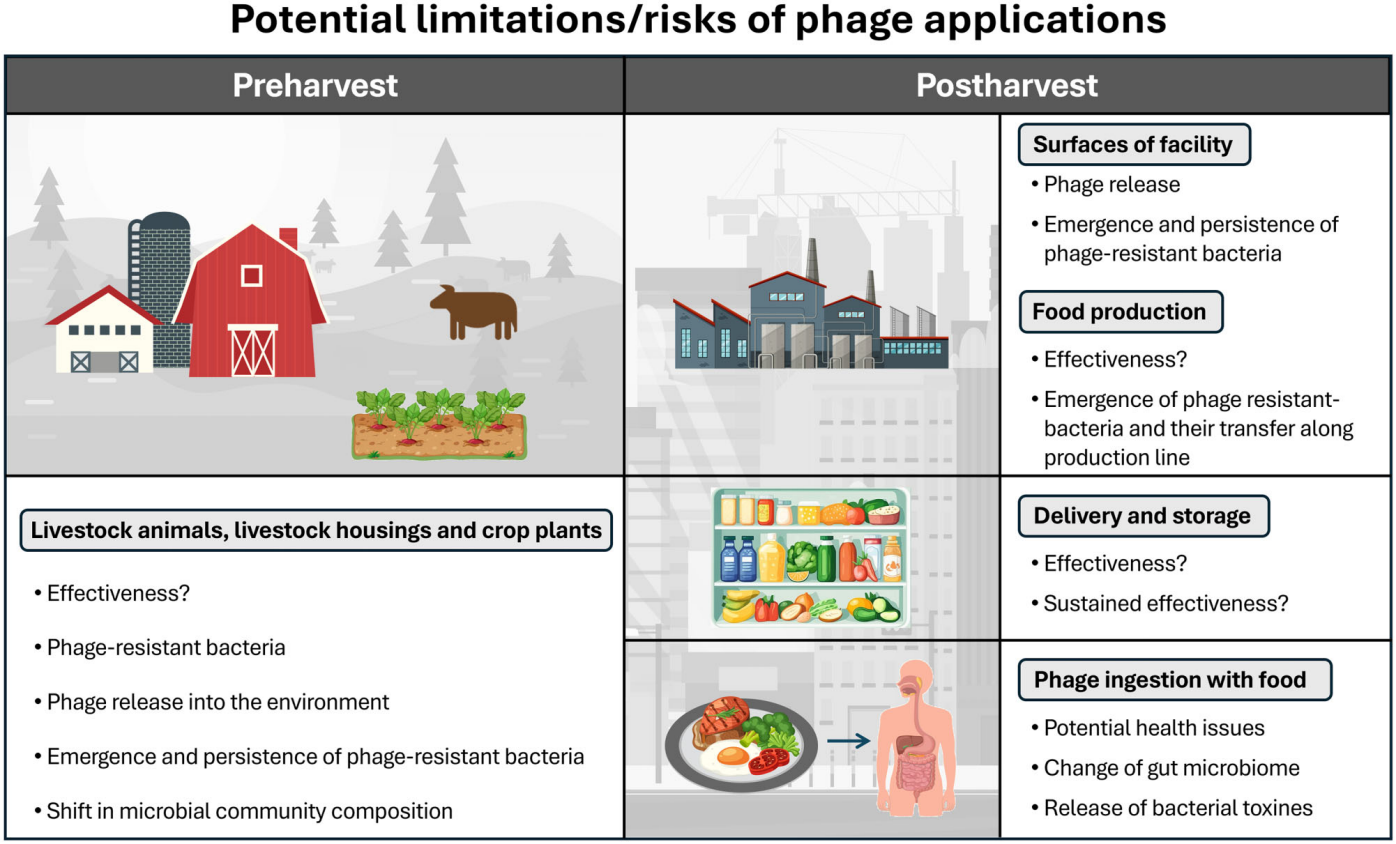
Potential limitations/risks of phage applications across different stages of food production. Left: Risks and limitations of phage applications in the preharvest sector (animal husbandry and crop cultivation): high phage loads on surfaces in livestock housings or on animals and crop plants and their possible release into the environment with unknown consequences for microbial communities; effectiveness may be compromised by limited host range, phage-resistant bacteria, or untargeted contaminations; widespread development of phage-resistant bacteria. Right: Risks and limitations of phage applications in the postharvest sector (processing and finished products): high phage loads on facility surfaces, promoting potential increase in phage-resistant bacteria and their persistence in facilities and production lines; effectiveness may be compromised by limited host range, phage-resistant bacteria or untargeted contaminations with direct consequences for food safety; phage ingestion via food with potential effects on gut microbiota and human health

Phages are often praised as a natural, biological approach for controlling pathogenic bacterial species on food and decontaminating production surfaces. Nevertheless, alongside the advantages, several considerations regarding the use of phages in food production are discussed (Table 1 and Fig. 2).

### Safety aspects during the production of phages

#### Appropriate phages and selection of bacterial strains for their propagation

Phages intended for application should not only be screened for strictly lytic activity, but also for a broad host range and efficacy under various environmental conditions. Their genomes must be fully sequenced to exclude potential lysogenic properties or other undesirable traits, such as antibiotic resistance or virulence genes, and to minimise the presence of genes with unknown function. To date, there is no evidence that virulent phages carry critical genes (Jebri et al. 2020). In accordance with good manufacturing practice, phage products should undergo quality control testing to verify their effectiveness.

It is the responsibility of manufacturers to carefully select the strains or serotypes used as host for phage propagation as well as to ensure the appropriate biosafety level, when fermenting pathogens. Laboratory strains often lack surface structures such as fimbriae, pili, lipopolysaccharides (LPS), capsule structures, or teichoic acids, which can be virulence factors but also serve as phage receptors necessary for infection. Therefore, virulence factors of host cells often cannot be avoided at the stage of phage production. However, pathogenic bacteria used for cultivation are unlikely to pose a risk of infection via treated food, since phages are separated from intact and lysed host cells as well as fermentation residues. Nevertheless, preference should be given to less virulent strains without antibiotic resistance to minimise the potential for undesirable gene transduction, even though generalised transduction is random and infrequent. The manufacturer is responsible for obtaining purified phages and for ensuring that phage cocktails are free from toxins or other harmful substances.

#### Modification/mutation of phages or bacteria during fermentation

Mutations in phage genomes can occur spontaneously during replication, with a mutation frequency higher than in bacterial genomes. Evolutionary pressure can arise as a result of cultivation conditions, such as temperature, pH, and bacterial host strain, potentially favoring mutant phages (Wichman et al. 2005). This, however, is typically avoided by maintaining optimal cultivation conditions (García et al. 2019). Due to the use of selected bacterial strains in pure culture, the host specificity of the phage is not subject to additional selection pressure. For example, the growth advantage of another bacterial species over the host strain, which could compromise phage specificity and thus efficacy for later application, is absent. It is unlikely that mutations affecting the safety of the application occur. Moreover, since bacterial cells are removed during phage purification, any mutations occurring in the host bacteria are irrelevant. Mutations in bacterial genes that impair phage infection or lead to phage resistance could theoretically reduce phage yield; however, such effects have not been observed in practice. The spontaneous mutation frequency in bacteria is approximately 10^–6^ per gene per generation (Lee et al. 2012). For example, a bacterial culture starting with 5 × 10^8^ CFU mL^–1^ would yield, at most, around 10^2^ bacteria mL^–1^ carrying a mutation in a specific gene (e.g. *E. coli* with 4.300 genes). Since multiple genes can contribute to phage resistance, the probability of resistance may increase. These genes may encode proteins involved in the synthesis of the bacterial capsule, affect cell wall or phage receptors structures or be necessary for successful phage replication (Labrie et al. 2010, Mutalik et al. 2020). Since not every mutation in these genes leads to phage resistance, the probability of resistant bacteria is considered rather low. After one hour of cultivation, bacteria that have acquired mutations conferring phage resistance constitute only a small fraction of the culture and are barely detectable (Lenski and Levin 1985). However, the situation differs for *Campylobacter* cultures, where phage-resistant hosts can often be detected at the end of a cultivation cycle due to capsule variants as described above (also see: “Relevant bacterial species”).

In general, a small number of successive bacterial cultures from initial cultivation to phage propagation may be necessary to maintain a low bacterial mutation rate. Continuous bacterial cultures for phage production have also been described (García et al. 2019), but a higher mutation rate is expected in such systems (Jurač et al. 2019), which could potentially affect phage yield. Any potential problems arising from mutations during phage production fall within the responsibility and interest of the manufacturer. They do not pose issues for the actual application, as the manufacturer is required to test and guarantee the efficiency of phage cocktails with a defined host spectrum.

### Safety aspects during the application of phages

#### Selection and effectiveness of phage cocktails

The selection of a phage cocktail, depending on the pathogenic bacterial species to be targeted, the type of food, and the stage of application along the production line (García et al. 2008, Cooper 2016, Cristobal-Cueto et al. 2021), is the responsibility of the user. Genera such as *Salmonella* and *Campylobacter* are commonly expected in raw produce, e.g. on poultry meat. Even if it is not known in practice whether contamination results from surviving cells after conventional processes (e.g. thermal treatment) or from secondary contamination within the production line, the user should be aware of which bacterial species are likely to occur and should rely on the manufacturer's instructions regarding application conditions. A consideration not supported by experimental evidence is that the survival of individual strains and serotypes of the pathogen outside the host range of a cocktail could represent a potential limitation of phage treatment, as such cells might, in principle, multiply on the food under favorable conditions. It could be considered whether applying multiple different phage cocktails, simultaneously or sequentially, might mitigate this potential risk. However, this approach remains largely speculative and would likely be constrained by practical factors such as cost and uncertainty regarding which strains and serotypes might persist. In the case of simultaneous application, dilution of each cocktail’s titre could reduce overall efficacy. It is also important to note that phage treatment does not replace proper cold-chain management or standard hygiene measures, which makes explosive multiplication of any remaining cells unlikely. Furthermore, reducing a portion of the contaminating strains and serotypes of a species would already improve food safety (Rosenquist et al. 2013). For surviving cells other than the targeted host cells, it can be assumed, due to the generally low cell numbers, that they do not gain a growth advantage and therefore do not pose an increased risk.

It remains unclear, whether the information provided on phage products is sufficient for the selection of effective phage cocktails, whether other precautions will be neglected by the user, leading to a proliferation of pathogenic bacteria, and whether the efficiency of the phage treatment should be monitored. There is also discussion about recording phage applications to take previous treatments into account when planning subsequent ones. In discussions on the safe use of phages, these points are mentioned as potential risks but fall within the scope of “good practice.” Some of these requirements are complicated by the fact that testing for contaminants is time-consuming and may not be routinely feasible. In practice, phage treatments tend to be used as a precautionary measure, even without knowledge of the contaminants. Users must be aware that phage treatment is not suitable for all types of food and does not completely eliminate the host strain. Studies using pre-inoculated food may overestimate the success of phage treatments. In practical applications, a surplus of phages is recommended, as indicated by the manufacturers' information on the concentration of the ready to use phage solution. Users must understand the limitations described above and should not rely on sustained or long-term activity against subsequent contamination, unless the manufacturer has explicitly verified and confirmed such extended activity (also see: “Conditions for phage applications” and “Phage/host ratio”).

#### Bacterial resistance to phages during application

The potential emergence of bacterial resistance to phages may pose a challenge of phage application (Hagens and Loessner 2010, Fernandez et al. 2018). Two scenarios are conceivable. First, resistance may arise spontaneously in the environment at an unknown point in time. Second, resistance may develop during phage treatment of food or of surfaces in production facilities. If such resistant cells proliferate in the environment or within the production line and subsequently contaminate food, they will no longer be killed by the existing phage or phage cocktail, which was selected for the expected non-mutated host. In such cases, phage cocktails adapted to the resistant variants may be necessary. Nevertheless, a phage cocktail, assuming a broad-host-range, would remain active against other strains and serotypes of a species. If there is an overlap in host specificity and comparable high titres of the individual phages are present, activity of the cocktail against the mutated strain may also be retained. The alternating use of different phage cocktails may help to reduce the likelihood of resistance development. However, the frequency at which target bacteria develop resistance is about 10^–4^–10^–7^ (Fernandez et al. 2018) and phage–host dynamics in growing bacterial populations are required for resistance to evolve (Sorensen et al. 2021). Combined with the typically low cell counts found on food, the occurrence of resistant bacterial cells on food is considered very unlikely (Hagens and Loessner 2010), unless host cells proliferate. As noted above, phages are not intended for use on highly contaminated foods. High cell counts may only arise if standard preservation measures, such as maintaining the cold chain or appropriate storage according to the manufacturer's good practice, have been neglected.

At high MOIs, increased resistance development of the host bacteria has been reported. This was observed when high titres of a single phage were used in inoculated chicken to reduce *Salmonella* (Atterbury et al. 2007), and also *in vitro* with *Enterococcus faecalis* at an MOI of 10 (Topka-Bielecka et al. 2021). Such conditions, high MOIs combined with bacterial proliferation, generally do not occur when phages are applied to food. In addition, the phage resistance observed in the cited studies was not stable. Many resistant isolates reverted to a phage-sensitive phenotype after several passages without phage exposure. This reversion may be due to a fitness cost associated with resistance (Meaden et al. 2015), whereby at least one physiological function is impaired, rendering the mutants less competitive in the absence of the phage (Topka-Bielecka et al. 2021).

Besides fitness, a decrease in virulence (de Melo et al. 2024) and, in some cases, in the antibiotic resistance of pathogenic bacteria (Chan et al. 2016, Li et al. 2024) under the influence of phages has also been reported. For example, the development of resistance to phage infection was associated with increased sensitivity to antibiotics in *L. monocytogenes* serovar 4b (Sumrall et al. 2019).

In therapeutic contexts, phages are also administered together with antibiotics. It has been investigated whether resistance to phages is associated with antibiotic resistance; however, this relationship has not yet been clearly demonstrated and is likely a multifactorial phenomenon within dynamically growing bacterial populations rather than the result of a specific change at a single resistance locus (Allen et al. 2017, McGee et al. 2023). A simultaneous development of antibiotic resistance or sensitivity with phage resistance is generally not expected, as phage receptors differ from antibiotic targets and resistance mechanisms against phages are unrelated to those of antibiotics. Nevertheless, phages have been isolated that rely on Tol C, an antibiotic efflux pump of the host cell. In such cases, mutations conferring phage resistance can also alter the host cell’s resistance or sensitivity to tetracycline (Burmeister et al. 2020). For food applications, a combined effect cannot be assumed, as antibiotics are present only in trace amounts, if at all, in certain foods, such as meat, and no actively growing bacterial cultures are expected.

#### Mobilisation of genetic elements and pathogenicity islands

Because of their use as therapeutics and in the food sector, virulent phages are under scrutiny regarding their potential to horizontally transfer genetic material or mobilise chromosomal elements (Doub 2021). Although selecting virulent phages is intended to exclude specialised transduction, the possible contribution of virulent phages to horizontal gene transfer via **generalised transduction** has been discussed (Waddell et al. 2009, Doub 2021).

Pseudolysogeny, the presence of a phage genome as a plasmid-like, non-integrated element, is typically associated with temperate phages and occurs under starvation conditions, when host metabolism is downregulated (Abedon 2009). For *Pseudomonas aeruginosa*, plasmid-like persistence of four virulent phages has been described that contributes to the development of host resistance. The long-term maintenance of extrachromosomal phage genomes within the host cell during pseudolysogeny has been suggested to play a potential role in **horizontal gene transfer** (Latino et al. 2016). Furthermore, during pseudolysogeny the phage is not lytically active (Venturini et al. 2022), which may be relevant for starving cells on food matrices.

For *S. enterica* serovar Typhimurium, a virulent phage capable of transducing bacterial DNA directed by a chromosomal *pac* site has been described. Pac-type-dependent phages are known to show variable efficiencies and properties in **mobilising chromosomal DNA segments** (Wolput et al. 2024).

Satellite systems, in which mobile genetic elements (satellites) coexist parasitically with helper phages, typically temperate phages, have also been described in connection with virulent phages. For *Vibrio cholerae*, it has been shown under laboratory conditions that infection with a virulent phage could activate a chromosomally located satellite associated with a temperate helper phage. This resulted in immediate phage resistance of the infected bacterium while the resistance determinant was disseminated as a plasmid (McKitterick and Seed 2018). Virulent-phage-influenced satellite systems have also been reported for *Streptomyces*, containing an extrachromosomal satellite (deCarvalho et al. 2023), and for a *Vibrio* species, containing a chromosomally integrated satellite (Barcia-Cruz et al. 2024).

For Gram-negative and Gram-positive bacterial pathogens, such as *E. coli* or *S. aureus*, pathogenicity island (PAI) helper-phage systems have been well described. In these systems, PAIs are mobilised and packaged into transducing particles assembled from proteins of a temperate helper phage (Gal-Mor and Finlay 2006, Christie and Dokland 2012). To the best of our knowledge, no virulent phages have been reported to date that act as helper phages or influence the **mobilisation of PAIs** in *E. coli* or *Salmonella* (Desvaux et al. 2020, She et al. 2025).

It is unlikely that mobilisation of genetic elements by virulent phages observed under experimental conditions (McKitterick and Seed 2018) plays a significant role during the application of phages on food or in production facilities. In contrast to natural settings, targeted bacteria are lysed through the use of phage cocktails, making the mobilisation of genetic elements improbable. Risks associated with virulent phages that may theoretically act as helper phages therefore appear to be more conceptual than practically verifiable.

Generalised transduction, pseudolysogeny and helper-phage systems may act as drivers of DNA transfer during long-term coexistence within bacterial communities in environmental settings. Whether the application of high doses of virulent phages in livestock and their release into the environment may significantly affect bacterial communities in this respect, and whether this raises safety concerns, remains unclear (see also: Release of phages into the environment).

#### Detection of remaining living cells of the target bacteria on foods

A potential problem with the use of phages on food is that bacteria in food samples may not be detectable using culture-based methods due to previous phage applications (Fister et al. 2019). If phages or phage cocktails are used in an intermediate stage of food production, they may be carried over unnoticed into the subsequent production steps and thus into food samples to be examined. Any remaining active phages could lyse the bacterial cells to be detected during the enrichment step of the culture-based detection methods and lead to false negative results (enrichment bias) (Muniesa et al. 2005). However, this scenario has neither been observed, nor scientifically demonstrated. A general assessment of a possible enrichment bias is difficult because considerable variations may occur in the strains and serotypes of a species involved, the amount and properties of the phages applied, the remaining number of active phage particles on treated food, and the characteristics of the food itself. Due to the generally low cell counts on food, e.g. 100 *Listeria* cells per cm^2^, phage proliferation remains limited, as there is only a small number of available host cells, even in the presence of high phage concentrations (around 10^7^ cm^–2^). Moreover, low storage temperatures and the resulting low division rates of the bacterial cells prevent exponential phage multiplication. In addition, the dilution steps included in culture-based detection methods, further dilute any intact phages that may be present. Consequently, infection and killing of the host cells in the enrichment step is unlikely. Nevertheless, no studies have directly investigated whether prior phage applications interfere with the culture-based detection of surviving bacteria in naturally contaminated food. In case of doubt, culture-independent detection methods may be used, such as quantitative real-time PCR- or ELISA-methods, which are often more sensitive than the cultural-based approaches (Dwivedi and Jaykus 2011, Liu et al. 2019). There is evidence that, when testing efficacy of phage applications in model systems with inoculated foods by culture based detection, remaining active phages lead to apparently higher killing rates (Chibeu and Balamurugan 2018, Dhowlaghar and Denes 2023). However, cell numbers, phage numbers, and detection methods for efficiency are not comparable to those used in routine food inspection. In the context of pathogen detection in food, it should also be noted that efforts are being made to establish phages or phage proteins for detecting of pathogens on food (Foddai and Grant 2020). Whether previous phage treatments interfere with such detection approaches remains an open question.

#### Release of phages into the environment

The potential release of large quantities of individual phage species used in the food sector is a topic of controversial discussion (Sommer et al. 2019). Released phages may influence the **natural balance between phage and bacterial species**. These concerns are, however, highly speculative, as it is difficult to generate robust data due to the complexity of the bacteria-phage ecosystem, which remains poorly understood (Czajkowski et al. 2019).

The amounts of **phages applied to food** are considered small compared to the quantity of naturally occurring phages (Vikram et al. 2021). Depending on the product, phages are applied at 10^7^–10^8^ PFU/g, although the maximum recommended amount is 10^9^ PFU/g (EFSA 2016). Extrapolated to the total amount of meat produced in the USA per year (approx. 45 billion kg), this would correspond to ∼4.5 × 10^22^ PFU, which represents only 0.000005% of the estimated minimum number of phages on earth (1 × 10^30^ PFU). In fact, the amounts of phages released into the environment are considerably lower, as concentrations of 10^8^ PFU/g are typically used, and by far not all phages reach the environment. Furthermore, a large proportion of phages are likely inactived after application, for example, through cooking or passage through the gastrointestinal tract. The use of **phages in animal housings and production facilities** along the production chain could result in the release of larger amounts of individual phage species into the environment (Meaden and Koskella 2013). For this reason, there is discussion about whether phages need to be inactivated after application (Sommer et al. 2019). However, such subsequent inactivation would contradict the original purpose of using phages for biosanitation, which is to avoid steps by means of chemicals, heat, or other treatments. Phages used in phage cocktails are of the class *Caudoviricetes* and typically lack a lipid envelope. Hence, decontamination agents that are membrane-active may be ineffective in inactivating such phages. However, systematic studies on phages that act against lactobacilli and are harmful to fermentation cultures have shown that many phages can be effectively inactivated using commercially available disinfectants (Hayes et al. 2017). If disinfectants, such as alcohols, (e.g. ethanol and isopropanol), aldehydes, acids and bases, chlorine and chlorine-releasing agents, or peroxides are applied subsequently, the additional benefit of a prior phage application may be questioned. There is concern that widespread use of phages could impact **bacterial populations in the environment**, potentially leading to the development of phage resistance on a larger scale (Meaden and Koskella 2013). Target bacteria may develop phage resistance to a greater extent than on food, as prolonged coexistence of phages and host cells and higher cell densities are expected in certain environments, such as biofilms (Hu et al. 2024). If resistant bacteria are subsequently transferred to food, they may no longer be susceptible to later phage treatment.

Based on current knowledge of coevolution, it is assumed that a variety of effective phages against the **newly emerged resistant mutant strain** or serotype can be isolated from the environment (Hudson et al. 2005, Artawinata et al. 2023). However, the search for and development of a highly lytic phage with a broad host range may be time-consuming and is not always straightforward (Sillankorva et al. 2012, Meaden and Koskella 2013, Glonti and Pirnay 2022).

Nevertheless, it is generally assumed that the release of large amounts of a few phage species acting against pathogenic bacteria does not pose a risk and may even be considered advantageous. Individual phage species can also naturally occur in high numbers when their host species is dominant within the bacterial population. Conversely, phage levels naturally decline once their target bacteria are no longer present and other bacterial species prevail, reflecting typical “predator-prey” dynamics (Chevallereau et al. 2022). The phages used in cocktails are naturally occurring phages, which further argues against major safety concerns. In addition, the pathogenic target species usually constitute only a small fraction of the overall bacterial community, making a fundamental shift in the natural bacteria-phage balance unlikely. Due to the limited data available, however, it cannot be conclusively determined whether the release of large quantities of individual, naturally occurring phage species has any environmental impact, but it appears unlikely.

### Release of bacterial toxins in food by phage treatment

There is an ongoing discussion regarding whether bacterial toxins (exotoxins or endotoxins) are released when bacteria are lysed during phage therapy (Lu and Koeris 2011, Dufour et al. 2017) or on food through phage application, and whether ingestion of these foods could lead to adverse health effects (Garvey 2022). Toxins produced in food by exotoxin-forming bacteria can cause intoxications when ingested. These bacteria may also enter the intestine via food, potentially leading to toxicoinfections through local toxin production (Noor 2019). To date, neither intoxications nor toxicoinfections have been described in connection with the use of virulent phages in food. Furthermore, ingestion of endotoxin-containing foods has not been associated with endotoxemia. Due to the limited knowledge available, current findings on endotoxins and on Shiga toxin, an exotoxin, are discussed below as example.

#### Phage treatment of food regarding endotoxins

Endotoxins are components of the cell wall of Gram-negative bacteria. They are heat-stable and weakly toxic. Endotoxins are released whenever Gram-negative bacterial cells lyse, but they are also shed during cell division. Since the intestinal microbiome is the largest reservoir for endotoxins in the body (André et al. 2019), they naturally occur in high quantities (approximately 1 g) in the intestinal lumen (Moreira et al. 2012) and in small amounts in the blood of healthy individuals (up to approximately 30 pg/ml blood plasma)^1^ (Nádházi et al. 2002, Erridge et al. 2007, Ghanim et al. 2009, Munford 2016, Brown 2019, Erlanson-Albertsson and Stenkula 2021).

Under normal conditions the presence of endotoxins in the intestinal lumen has no effect on human health (Moreira et al. 2012). Endotoxins become critical only when they enter the bloodstream in large quantities (up to 0.8 ng/mL), which is called endotoxemia. This can result from a compromised intestinal barrier, for example during intestinal infections, or from systemic or other bacterial infections.

Diet-related changes in the gut microbiome, caused by shifts in the balance of endotoxin- producing and non-producing microorganisms, have been discussed as a potential cause of dietary endotoxemia (Kelly et al. 2012). However, data are lacking on the extent to which oral ingestion of endotoxin-forming bacteria or released endotoxins via food contributes to endotoxemia. Gram-negative bacteria with the potential to release endotoxins occur naturally in food. Even after processing steps that may cause cell lysis, such as heat treatment, the toxins remain intact. Given the already high content of free endotoxins in the intestinal lumen, oral uptake of these bacteria or their endotoxins should be negligible. Due to the expected low cell counts of pathogenic bacteria in food, the level of endotoxin release caused by phage treatment or other preservation methods, should not pose an increased risk. .

#### Phage treatment of food regarding exotoxins

One of the largest *E. coli* O104:H4 infection outbreaks worldwide caused by a strain belonging to the STEC (Shiga toxin-producing *E. coli*)^2^, occurred in Germany in 2011. About 22% of the infected individuals developed “*E. coli*-induced hemolytic uremic syndrome,” a potentially fatal form of kidney damage, and more than 50 people died (Burger 2012). The expression of Shiga toxins takes place in the intestine during bacterial multiplication. The toxins are released when the prophage switches from the lysogenic to the lytic cycle (Wagner et al. 2002, Herold et al. 2004). They are then taken up by the intestinal epithelial cells through receptor-mediated endocytosis (Schüller 2011).

Phage treatment is considered a promising method to reduce exotoxin-producing *E. coli* strains to increase food safety (Pinto et al. 2020, Necel et al. 2021). On the other hand, there is concern that if bacterial cells are lysed in large numbers on food, they could release sufficient amounts of exotoxins to cause harmful intoxication upon ingestion. Intoxication by Shiga toxins released directly on food has not yet been described. It is generally assumed that Shiga toxins enter the bloodstream only after bacterial infection of the intestinal mucosa leading to bloody diarrhoea (Butler 2012). It remains to be clarified to what extent the uptake of Shiga toxin from food into the intestinal cells can be compared with *E. coli* infections of the intestine, which may be necessary for mucosal uptake of the exotoxins.

There is also discussion about whether Shiga toxin-encoding, temperate phages can be released on food by virulent phages targeting the corresponding *E. coli* strains and whether these temperate phages could infect other *E. coli* strains in the intestine after oral ingestion. However, for infection to occur, the intestinal strains would already need to carry genes for additional virulence factors (Muniesa et al. 2012, Rodríguez-Rubio et al. 2021), making this route unlikely. Stable acquisition of Shiga toxin-encoding lambdoid phages is rarely observed (Tozzoli et al. 2014) and no data are available regarding the food sector. Nevertheless, the history of Enterohemorrhagic *E. coli* (EHEC) infections worldwide shows that evolutionary successful and stable new combinations of virulence factors, including prophages, can occur. Examples include the “big five” serogroups of epidemiologically significant EHEC that have caused sporadic outbreaks worldwide (Eichhorn et al. 2015).

There is evidence that, under laboratory conditions, infection of *E. coli* O157:H7 with virulent phages resulted in a reduced release of both Shiga toxin and Shiga toxin-encoding phages (Howard-Varona et al. 2018). However, since the outcome of such experiments depends on numerous parameters, it cannot be reliably concluded that using virulent phages in food poses no risk of increased toxin release. On the other hand, virulent phages could serve as a method to eliminate exotoxin-forming bacteria on food as efficiently as possible. Compared to this, toxiinfection or intoxication by toxin excreters multiplying in food or the intestinal tract poses far greater risks.

The chromosome of *S. enterica* contains various prophages (Thomson et al. 2004, Wahl et al. 2019) that can be carriers of virulence factors like exotoxins. There is further discussion about whether routine phage use against *Salmonella* in food could lead to the release of virulence factors and temperate phages and subsequently to horizontal gene transfer between different *S. enterica* serotypes (Worley et al. 2018, Bawn et al. 2020) potentially resulting in serotypes with altered virulence (Brüssow et al. 2004).

Even though phage cocktails against *S. aureus* have not yet been established, this species may also harbor phage-encoded exotoxins that could be released (Argudín et al. 2010, Hennekinne et al. 2012).

Finally, it should be noted that conventional methods already used to preserve food and improve food safety would have similar consequences in the presence of toxin-producing bacterial species.

### Influence of food-borne phages on the gut microbiome

#### Naturally occurring phages in the intestine and on food

Phages are part of the natural gut microbiome (the phageome) and are ingested daily in large quantities with food (Tsuei et al. 2007, Shkoporov and Hill 2019). They naturally influence the **microbial balance in the gut**, although it cannot yet be conclusively assessed how this affects human health (Gogokhia et al. 2019, Sweere et al. 2019). The total number of phages in the intestine is estimated to be up to 10^15^ (Shkoporov and Hill 2019). By comparison, the consumption of one portion of phage treated food (100 g) would result in the ingestion of approximately 10^9^ phage particles, most of which are likely inactive.

Phages are as diverse as the bacterial species in the gut. For example, an analysis of 28 060 human intestinal metagenomes identified 142 809 non-redundant phage genomes (Camarillo-Guerrero et al. 2021). The proportion of temperate phages is often considered high, although such estimates mainly reflect prophages identified within bacterial genomes rather than freely circulating virions (Pei et al. 2024). Freely detectable temperate phages are likely far less abundant than virulent phages (Shkoporov and Hill 2019, Avellaneda-Franco et al. 2023).

The **intestinal phageome** is thought to influence both the maintenance of intestinal health through the regulation of the microbiome and the development of disease via alterations in the bacterial population (Carding et al. 2017). Phages have been reported to accumulate in the mucus layer of the intestine and to contribute to controlling the bacterial load on the intestinal mucosa. Uptake of certain phages by the intestinal epithelium has also been proposed (Nguyen et al. 2017). It is assumed that phages can cross the intestinal mucosa via endocytic uptake by epithelial cells and reach the lymph and bloodstream. Moreover, it has been suggested that certain phages interact with eukaryotic tissues, can be degraded by eukaryotic cells, and that eukaryotic cells may even transcribe phage genes. Phages have been described as natural immunomodulators that interact with the mucosal immune system and, after entering lymph nodes, the bloodstream, and various organs, influence both the innate and adaptive immune responses. Through these interactions with various immune cells, they are believed to contribute to the maintenance of immune homeostasis (summarised by Barr 2017, Jariah and Hakim 2019, Dery et al. 2021). In an experimental mouse model, bacteriophages were shown to exacerbate colitis via Toll-like receptor 9, providing further evidence that naturally occurring phages may modulate immune systems, albeit triggering unwanted signaling in this case (Gogokhia et al. 2019). However, it is unlikely that food borne virulent phages contribute to such effects. There is also no evidence that phages themselves possess virulence against human or other eukaryotic cells and act as human pathogens (Kutateladze and Adamia 2010). It is conceivable that certain structures of the phage capsid interact with eukaryotic cells without “infecting” and lysing them.

Only few quantitative data are available on **naturally occuring phages in food**. However, it is evident that wherever bacteria are present on or in food, phages can also be found and that coevolution occurs. This is particularly true for products preserved by fermentation, a long-established traditional method (Vogel et al. 2011). Only a limited spectrum of phages has been determined on selected foods to date. For *Enterobacteriacae* on raw meat, poultry meat, fish, and seafood, phage levels of up to 10^3^ PFU/g have been detected. For *Pseudomonas, Leuconostoc* and *Enterobacteriaceae* on refrigerated foods such as raw ground beef, sausages, raw milk, and oysters, levels up to 10^7^ phages/g have been observed (EFSA 2009). Other studies found less than 10^3^ PFU/100 g in finished sausage products (EFSA 2009). However, the true level of naturally occurring phages in food is likely higher, as only a small fraction is detected when phage numbers are measured via the lysis of specific host bacteria.

#### Ingestion of phages through treated foods

In therapeutic applications, phages are used to positively influence and support the natural gut microbiota (Rasmussen et al. 2020, Dahlman et al. 2021), as well as to specifically reduce undesirable bacterial species in order to combat infections (Brussow 2017, Abedon et al. 2021). Numerous publications have investigated the dissemination and efficacy of phages primarily after oral ingestion, but also after inhalation, injection or skin application (summarised by (Cui et al. 2019, Dabrowska and Abedon 2019)). Systemic uptake of oral ingested phages across the intestinal barrier is very low. During gastric passage, most of the phages may be inactivated by acid pH and/or digestive processes. Oral phage therapy therefore often involves neutralising the gastric juice or administering the phages in encapsulated form (Rotman et al. 2020). Studies on the tolerability of orally ingested phages typically span only a few days. Single doses usually range from 10^7^ to 10^9^ phage particles, with 10^6^ particles considered a low dose (Sarker et al. 2012, Sarker et al. 2016, Sarker et al. 2017, Febvre et al. 2019, Gindin et al. 2019). In almost 70 individual case reports of therapeutic phage use in humans, phages were administered orally in seven cases, for periods ranging from few days up to 16 weeks, without any observed side effects (Suh et al. 2022). In two studies in rats, impaired intestinal permeability and increased levels of pro-inflammatory cytokines were reported following oral administration of phage cocktails intended for food application, raising concerns about the safety of orally ingested phages (Tetz and Tetz 2016, Tetz et al. 2017). However, both studies exhibited substantial methodological flaws, including small sample size (*n* = 5) and the absence of appropriate controls.

Whether the chronic oral intake of large quantities of a few phage species used in food has harmful effects on the human intestinal microbiome or phageome, and thus on health, has not yet been conclusively investigated in human studies. The pathogens *Listeria* and *Campylobacter* spp. are not part of the natural gut microbiome, and their lysis in the intestine would be expected to have positive effects. *E. coli*, on the other hand, are prevalent commensals in the human intestine, while pathovars of this species occur on animal and plant foods. This raises the question of the extent to which phages effective against *E. coli* may also affect coliform bacteria in the intestine (von Strempel et al. 2022). For phage selection, specificity for STEC would be advantageous, a carrier of prophages that encode Shiga toxins (Rodríguez-Rubio et al. 2021). There are specific phages that exclusively infect O157:H7 serovars prevalent in the food sector, the main representatives of EHEC^2^ strains (Mozaffari et al. 2022). In addition, studies have shown that the oral administration of phages targeting *E. coli* does not reduce the number of naturally occurring *E. coli* in the intestine (Bruttin and Brussow 2005, Sarker et al. 2017). A further indication that orally ingested phages may not have adverse effects is the absence of corresponding reports from countries in which phages have long been used in the food sector. Aspects of the gut phageome, the need of narrow-spectrum antimicrobials, and the potential benefits of phages are discussed elsewhere (Mills et al. 2017, Pinto et al. 2020, Hassan et al. 2021).

### Allergenicity

As mentioned above, phages are ubiquitous and are ingested daily with food, without any adverse health effects having been described to date (Hagens and Loessner 2010). So far, there are also no indications that the use of phages on food poses an allergenic risk to consumers. No immunological reactions associated with phage therapy have been observed after oral administration, and only absent or mild antibody responses have been reported after local injection (Kakasis and Panitsa 2019). Phage proteins on their own do not appear to be allergenic. In fact, *in silico* analysis of the primary structure (amino acid sequence) of phage proteins revealed no similarities to known allergens (Ramirez et al. 2018). Moreover, phages appear to exert anti-inflammatory action and have been proposed for the treatment of specific cases of allergic disorders due to their significant immunomodulatory properties, while allergic reactions to phage administration seem to be negligible (Górski et al. 2018). This is supported by the fact that viruses, including phages, tend to promote an adaptive immunological response involving Th1 lymphocytes, whereas allergies in particular are subject to an immunological pathomechanism involving Th2 lymphocytes (Klimek et al. 2018).

## Legal aspects of phage application in the food sector

### Possible classification of phages used on foods

#### Classification of phages as processing aid

Processing aids are substances used in the processing of food or food ingredients for technological reasons. They are not consumed as food but, like their degradation products, may be present in the final product as unavoidable residues, provided they are harmless to health and have no technological effect on the final product.^3^ Processing aids are not subjected to labeling on food packaging.

When phages are used as processing aids during food production, by definition, only inactive phages should be present in the final product and only in low, unavoidable quantities. The fact that phages are viruses that become active within bacterial cells complicates their classification as processing aids. Despite this, they are already used as such in many countries outside the EU. A technological effect on the final product cannot be fully excluded due to the potential activity of phages that may remain on food. The classification of phages (such as the preparation Listex) as processing aid under food law is currently under discussion among EU Member States (COM Working Group). As a result of these deliberations, it has been noted that the majority of the participants does not consider or accept phages as processing aids.

#### Classification as food additive

Food additives are substances added to a food for technological reasons during manufacture, processing, preparation, treatment, packaging, transport, or storage and may or may not possess nutritional value. As a rule, they are neither consumed as food nor used as a food ingredient. Food additives must be approved^4^ and are subjected to mandatory labeling.

When used as food additives, phages would become components of the finished food product, as removal is not foreseen for additives. No distinction would be made between active or inactive phages, since inactivation is also not foreseen for food additives. Additives are categorised into different functional classes, with phages falling under preservatives due to their intended effect. Preservatives are defined as substances that prolong the shelf life of food by protecting it from the harmful effects of microorganisms, and/or inhibit the growth of pathogenic microorganisms. Due to their high specificity for certain bacterial species, phage cocktails cannot be equated with conventional preservatives. Instead, phages enhance the safety of specific foods by reducing the pathogen species typically associated with them (e.g. *Listeria* in dairy products). As EU Member States are currently discussing whether microbiological cultures used for preservation should be classified as food additives, the classification of phages for preservation purposes would likewise be conceivable. However, since the outcome of the discussion on microbiological cultures is still pending, a decision regarding phages is not expected in the near future. An important requirement would be that each phage/phage cocktail undergoes individual approval, including proof of specificity, efficacy, duration of action, and clearly defined possible applications based on its host range.

#### Classification as decontamination agent

In the case of agents used to remove microbial surface contamination from food of animal origin, special regulations apply in accordance with Regulation (EC) No 853/2004. Currently, only drinking water is permitted for use on carcasses, whereas lactic acid is additionally allowed on beef carcasses.

To date, the European Commission has not approved phage products as a means of reducing bacteria in RTE foods and has banned their marketing in the EU as processing aids for the purpose of inactivating pathogens on, for example, carcasses. One justification is the concern that phages might be used as a substitute for hygiene measures rather than as a complementary tool. While rinsing with water removes contaminants in general, phage treatments selectively target only specific bacterial species.

#### Equating phages with microbial starter cultures

There are considerations as to whether specific phages could be regarded as naturally occurring, microbiologically active particles that are harmless to health, similar to bacteria used as starter cultures in food production. Such starter cultures are traditionally used and, as an exception to the additive ban, are permitted without authorisation (Vogel et al. 2011). Although the natural presence of phages in starter cultures may argue for their use as harmless agents, these phages are unintended and uncharacterised particles accompanying starter cultures, and they do not contribute to the fermentation process. Since the targeted use of phages for decontamination of food is not comparable to the purpose of starter cultures, equating them with starter cultures is not admissible. Consequently, phage application on food cannot be justified as a traditional use that would not require authorisation.

#### Approval of phages as a “Novel Food”

There are also proposals to categorise phages intended for application on food as “Novel Food” within the meaning of Regulation (EC) No 258/97. However, the use of phages as “Novel Food” requires a safety assessment and authorisation, as they were not used to a significant extent before the cut-off date of 15 May 1997, which is a requirement for the use without authorisation. It should also be noted that phages are added to foods to achieve an effect and are not considered a food in their own right. Any conventional food treated with phages would therefore become a novel food and would require authorisation. This approach could be considered for individual foods under special conditions, but it is not comparable to the widespread use of phages.

#### Phages used as biocides on surfaces in food processing facilities

Substances and mixtures used to remove microbial surface contamination from work and production surfaces, or from vessels and supply lines, are covered by a regulation on the use of biocidal products, including their provision on the market.^5^ The application of phages in the food sector, particularly against biofilms on food contact surfaces, is considered to have a high potential (Bhandare and Goodridge 2021), but has not yet been approved.

### EFSA statements on the use of phages in the food sector

There is currently no legal regulation in the EU governing the use of phages in food production. In 2009, EFSA released a statement on behalf of the European Commission regarding the use and mode of action of phages on food of animal origin (carcasses, meat, or dairy products), concluding that insufficient data were available to assess efficacy and duration of action, including activity against a subsequent contamination in finished food (EFSA 2009). The EFSA opinion noted that phages may have significant potential in selected applications, depending on the bacterial species and foodstuff (EFSA 2009). In two EFSA opinions on a phage product containing a single phage targeting *Listeria* on raw fish and on RTE foods containing meat, fish or dairy products, EFSA concluded that the data demonstrating widespread elimination of *Listeria* were insufficient (EFSA 2012, EFSA 2016). A stated risk was the survival of phage-resistant *Listeria*, which are supposed to be naturally present to a certain extent. Additionally, EFSA requested research on the efficacy of the phage product on RTE foods naturally contaminated with *Listeria* and investigations into the molecular mechanisms underlying the alleged phenomenon whereby bacteria resistant to certain antibiotics became sensitive upon acquiring resistance to the used phage. In 2021 and 2023, EFSA concluded that the phage cocktail Bafasal® containing four phages against a specific *S. enterica* strain, is considered safe for birds and poultry when used as a feed additive. Furthermore, poultry fed with treated feed was deemed safe for the consumer (EFSA 2021). Nevertheless, EFSA noted that data on the efficacy of the phage cocktail in any avian species are insufficient, that the product only addresses *Salmonella* spp. contamination in the intestine and feces, and that its potential to reduce the *Salmonella* spp. contamination in poultry carcasses and/or the environment is limited (EFSA 2021, EFSA 2023). In the event that the cocktail is approved, EFSA also recommends a post-market monitoring plan to address the potential selection and spread of resistant variants of *Salmonella* to Bafasal® (EFSA 2023).

According to the current EFSA assessments, the limited host range of phages or phage cocktails, as well as their insufficient efficacy and the unproven persistence of phage activity, are considered the main risks of phage applications on food, particularly if established decontamination methods were to be replaced (EFSA 2012, EFSA 2016).

The European Court of Justice has repeatedly rejected actions by manufacturers against these decisions. In 2019, in the absence of a harmonised European legal framework, a court ruling left it up to individual Member States to allow food producers to use phages against *Listeria* as processing aids (excluding their use as food additives) under national legislation^6^. It was also noted that many EU Member States reject the use of phages to treat food (Curia.europa.eu, paragraph 55: *From the discussions with Member States, it became clear that there was too much opposition against Listex^™^ P100 to allow the application for its approval any possibility of obtaining political support. In that regard, by letter dated 19th February 2018, the Commission* (Note: EU-Commission, represented by B. Eggers, W. Farrell and I. Galindo Martín, acting as Agents) *informed Micreos Food Safety of its decision that it did not intend to pursue the evaluation of the application file*)^7^. In the Netherlands, phage cocktails targeting *L. monocytogenes* are now authorised as processing aids for use in food production, including certain raw meat, poultry and cheese production and for the decontamination of production facilities. At the end of 2025, EFSA released the draft “EFSA Draft … on the safety and efficacy of substances for the removal of microbial surface contamination of foods of animal origin intended for human consumption” for public consultation,^7^ suggesting that phages may be permitted as decontamination agents on food of animal origin in the EU in the coming years.

### Authorisation and classification of phages in non-EU countries

In the USA, the FDA issued GRAS notifications for phage cocktails developed by companies, each assigned a GRN number (Table 2). These notifications indicate that they are considered safe for human consumption. Under the GRAS designation, phages may generally remain in finished food products according to the conditions outlined in the GRAS notices, without requiring their removal as would normally be expected for processing aids. The effectiveness of the phages is not evaluated as it is by EFSA; only their safety is assessed. For regulatory clarity in meat and poultry production, some of these products are evaluated and listed by the Food Safety and Inspection Service (FSIS) of the United States Department of Agriculture (USDA) under Directive 7120.1 as processing aids for specific applications (*e.g*. ListShield™, PhageGuard E™, SalmoFresh™). This approval does not alter the GRAS conditions; therefore, removal of the phages from the finished product is not required. Only ListShield™ is formally approved by the FDA as a food additive under 21 CFR § 172.785, which allows its direct application to finished products with regulatory certainty. All other phage products are, in practice, used within a regulatory grey zone when present in finished products, relying solely on their GRAS status.

Health Canada grants approvals in the form of Letters of No Objection (LONO) or interim LONO (iLONO). About six phage cocktails are approved as processing aids; all of these have GRAS status. In Israel, five GRAS-classified phage cocktails are approved, and in Australia/New Zealand, two. In Switzerland, two phage cocktails with GRAS-status targeting *L. monocytogenes* are listed as processing aids for cheese production in the FDHA regulations.

The Scandinavian countries, which are only partially EU members (Denmark, Sweden, Finland, but not Norway and Iceland), sometimes assess food-related matters jointly, independent of the EU’s EFSA. Currently no approvals exist for phage cocktails for food in these countries, although Norway is evaluating their use in aquaculture.

In the UK, there is currently no established regulatory approval for the use of bacteriophages on foods, and a uniform authorisation pathway for food-related phage applications is still lacking. Research initiatives and pilot projects involving bacteriophages in the food sector exist, but commercial, regulated use has not yet been implemented.

Similarly, in South America, there is currently no broad regulatory approval for the use of phages on foods. Brazil has approved a phage preparation for use in animal feed, similar to EFSA in the EU (e.g. Bafasal^®^). Chile has established a commission to develop approval criteria and guidelines for phage applications. Argentina has laid the regulatory groundwork for phage approval but has not yet approved any products. In Colombia, Peru, Ecuador, Uruguay, Paraguay, and Venezuela, test trials with phages have been conducted for certain applications (e.g. aquaculture), but no nationally documented approvals exist. In Russia and China, the focus of phage approvals is on therapeutic applications in human and animal health rather than on food use. One Chinese company produces a phage cocktail with GRAS status (by FDA) against *S. enterica*. Scientific discussions and research on phage use in the food sector exist, but no regulated approval mechanisms are in place. In the Arab countries, as well as in other countries such as Japan, Korea, and India, there are no publicly documented approvals for the use of bacteriophages on foods.

Almost all globally approved phage cocktails have GRAS status from the US FDA (Table 2), making it the leading authority in this field. While the FDA has notified most phage products as GRAS, with no requirement for their removal, authorities in other countries classify the same products as processing aids, where the obligation for removal is subject to national regulations. The USDA’s approval of phage products as processing aids serves primarily as an additional regulatory tool to ensure their proper application on carcasses, raw meat, poultry, and other animal-derived products.

The use of a phage cocktail approved as a processing aid on RTE foods without subsequent rinsing, would violate EU law. Conversely, if the product is approved as a food additive, under EU regulations these RTE products would need to be labeled as containing phages. These inconsistencies illustrate the regulatory challenges and uncertainties that complicate the approval of phage products in the EU.

## Evaluation criteria of phage use in the food sector

The **transfer of DNA** is minimised by selecting virulent phages, which the manufacturers and the regulatory authorities must ensure. The most reliable way to exclude lysogeny and other safety-relevant properties such as genes of virulence factors is to sequence the isolated phages.

Because of the **restricted host range**, suppliers should test the phage cocktails on the broadest possible spectrum of strains and serotypes of the target species for which the phage cocktails are intended to be used. The food manufacturers and users must have precise knowledge of the safety-relevant bacterial species and strains present in their specific field of application. This also requires regular monitoring. Where indicated, further testing is recommended to determine whether other strains and serotypes of the target species have emerged, so that the phage cocktails can be adjusted accordingly.

The conditions required for the **effectiveness of phage products** must be clearly specified. In particular, the characteristics of the food matrix need to be considered. For each phage product, it should be clearly established how long the phages remain active under specific application and storage conditions, including the minimum exposure time and the conditions that allow prolonged phage activity. It may also be useful to identify, which foodstuffs or food groups could particularly benefit from phage applications. Complete (100%) elimination of pathogenic bacteria cannot be achieved with phage cocktails, or any currently approved method used along the food chain, except sterilisation procedures. This incomplete elimination does not constitute a particular risk of phage use; it is to be expected. As a rule, the use of phages can only achieve an additional reduction of the target bacteria and should not be used to replace established methods.

The occurrence of **false-negative results in culture-based food controls** cannot be completely ruled out. Different culture-based methods can be influenced by various factors, such as the amount and properties of the used phages, the remaining number of active phage particles, the characteristics of the food, and the bacterial species involved. Application-oriented research would therefore be helpful to develop standards that rule out false negative results in culture-based methods. However, culture-independent methods are increasingly being used for the detection of pathogenic bacteria.

Oral ingested phages do not pose an **allergenic risk**. Endotoxins and exotoxins that may be released during phage production are also not considered to pose a risk. Whether the release of **bacterial toxins** on phage-treated foods or in smear water of foods affects health has not yet been described but cannot be completely ruled out. Similarly, increased lysis of bacterial cells by phages ingested through treated foods, and the possible subsequent release of toxins in the intestine, has not been reported to affect health, but cannot be ruled out either.

Very little research has been conducted to date on the impact of phages on the **composition of the gut microbiome**. Based on the naturally occurring daily intake of phages, existing studies on therapeutic oral phage administration (Liu et al. 2021, Duan et al. 2022) and the worldwide use of phages in the food sector, there are indications that no health hazard is expected. However, further studies are needed to confirm that chronic oral intake of phages used in food has no impact on human health.

The **development of phage resistant bacteria** can occur when phages are used in prolonged coexistence with metabolically active target bacteria under certain circumstances. It would be useful to document the phage applications, particularly when treating intermediate products or facilities in the production chain. Emerging resistant bacteria could be carried over into the next processing step, potentially influencing the efficacy of subsequent phage applications. To prevent the emergence and spread of resistant bacteria, application standards for surface treatments should be developed. Regular monitoring of the treatment's effectiveness can help to adapt phage cocktails to any potentially emerging resistance, if necessary.

When applied to food, the **release of phages into the environment** is considered negligible due to the relatively small quantities used and the subsequent processing or consumption of the food. The situation differs in the case of large-scale surface applications. Here, the potential development of resistance in target species within the environment should be considered as a possible limitation for phage use.

## Conclusion and proposals for further research

The various limitations discussed as safety-relevant for phage use in the food sector can be weighted differently. Almost all potential limitations and possible risks can be reduced or avoided through quality management system. This system should include standard instructions for phage isolation, production and use, taking into account the field of application, the target bacteria, the food items, and the conditions for effectiveness. The management system should also include continuous monitoring of host specificity and phage cocktail efficacy, as well as instructions to verify efficacy. Individual authorisation of each phage cocktail is fundamentally necessary. In this context, a QPS system (Qualified Presumption of Safety), as used for the safety assessment of microorganisms in food production, is recommended. The successful use of phages in the food industry outside the EU indicate that these challenges are manageable. Potential effects on the gut microbiome and possible disadvantages for human health should, however, be kept in mind.

### Points to consider and to explore:

1. To further improve sequence analyses of complete phage genomes as a means of safety assessment, efforts should be made to determine the function of unknown genes of phages that are used or are intended for use.
2. It should be investigated whether, and if so to what extent, phage resistance develops in the relevant bacterial species under typical use conditions in the food sector, particular for the treatment of work surfaces. It should also be clarified whether this constitutes a risk or only a limitation (e.g. due to a constant, slight reversion to the phage-sensitive phenotype), and which conditions could prevent the development of resistance.
3. It should be clarified whether endotoxins released by the lysis of Gram-negative bacteria in food or in the intestine by applied phages may increase the endotoxin content of the intestinal lumen and blood, particularly for sensitive persons.
4. If relevant, it should be investigated whether lysis of lysogenic bacterial species in or on foods during phage treatment may release exotoxins or exotoxin-encoding, temperate phages and whether the consumption of these foods could have negative health effects.
5. Although investigation is possible only with selected food models, it should be studied whether active phages remaining in food samples to be tested may influence the culture-based detection of pathogenic bacteria. It should be noted that there is great variability in bacteria, phages, and food matrices. It should also be studied to what extent culture-based methods might be replaced by non-culture-based methods.
6. Further animal and human studies should be conducted to investigate whether repeated ingestion of large quantities of individual phage species applied to food has a negative impact on the gut microbiome, gut function, and overall health, or whether it may have a positive impact by targeting pathogens that are not normally part of the gut microbiome.
7. If the hypothesis that some specific phage species also interact with eukaryotic organisms or cells, which is based on a small number of initial studies, is confirmed, this interaction should be ruled out for phages used in the food sector.
8. It should be investigated whether phages that are added to (solid) foods are able to reduce a subsequent recontamination by the host bacteria.
9. It should be verified whether phage applications contribute to sustainability in food production and supply, even if it is predominantly recommended as an additional measure.

## Supplementary Material

fuag002_Supplemental_File

## Footnotes

1
https://www.fda.gov/media/152868/download Limulus Amebocyte lysate assay (LAL test)], for comparison: 1 ng LPS *E.coli* 055:B5 corresponds to 10 EU, https://acila.com/acilaeng/produkte/endotoxine/endotoxinfs.htm)

2 causing symptoms of hemorrhagic colitis or the hemolytic uremic syndrome

3 Regulation (EC) No 1333/2008 of the European Parliament and of the Council of 16 December 2008 on processing aids, Art. 3, para. 2(b)*

4 Regulation (EC) No 1333/2008 of the European Parliament and of the Council of 16 December 2008 on food additives, Art. 3, para. 2(a)* *https://eur-lex.europa.eu/legal-content/en/TXT/?uri=CELEX%3A32008R1333

5
https://eur-lex.europa.eu/eli/reg/2012/528/oj

6
https://curia.europa.eu/juris/document/document.jsf?mode=DOC&pageIndex=0&docid=236221&part=1&doclang=EN&text=&dir=&occ=first&cid=6675830 (paragraph 131 and paragraph 55)

7 The public consultation period has ended; the document is now only accessible here: https://cot.food.gov.uk/EFSA%20Draft%20Guidance%20for%20Public%20Consultation%3A%20submission%20of%20data%20for%20the%20evaluation%20of%20the%20safety%20and%20efficacy%20of%20substances%20for%20the%20removal%20of%20microbial%20surface%20contamination%20-%20Introduction%20and%20Background

## Glossary

Bacteriocins: Antibiotic peptides or proteins produced by certain strains of bacteria that inhibit the growth of other strains of bacteria that are usually closely related.
Biocontrol: Control of animal or human-pathogenic bacteria by biological means.
Biopreservation: Preservation of food and other products threatened by spoilage through biological processes (also bioconservation)
Biosanitation: Decontamination of non-biological material not intended for consumption, such as work surfaces or tools, by means of biological aids.
Capsid: Viral envelope that encloses the genome of the bacteriophage; also refers to phage head consisting of viral envelope and enclosed phage genome.
Capsule: Additional jelly-like protective layer of bacterial cells outside the cell wall, composed of polysaccharides.
CFU: Colony forming unit: this is a bacterial cell that becomes a bacterial colony through cell division, the cells of which are genetically identical.
EHEC: Enterohemorrhagic *E. coli*
Endolysins: Hydrolytic enzymes encoded in the phage genome and synthesised in the bacterial cell during phage propagation.
ESBL: Extended-Spectrum-Betalaktamase
ETEC: Enterotoxic *Escherichia coli*
Fimbriae: Filamentous cell appendages of bacterial cells for adhesion to cell surfaces.
Flagella: Thread-like structures (cell organelles) on the surface of bacterial cells that can cause the bacterial cell to move.
GMP: Good manufacturing practice
Gram-negative: The staining of the bacterial cell wall according to H.C. Gram with certain basic stains (e.g. gentian violet) is negative, i.e. it is washed out again by ethanol, as there are no teichoic acids and only a single-layer murein shell and the stain is not retained in the cell wall.
Gram-positive: The staining of the bacterial cell wall according to H.C. Gram with certain basic stains (e.g. gentian violet) is positive, i.e. it cannot be washed out again by ethanol, as teichoic acids and a single-layer murein shell retain the stain in the cell wall.
Gut microbiome: The totality of all DNA sequences of the microorganisms found in the intestine.
Gut microbiota: The totality of all microorganisms in the intestine.
Integrase: Enzymes for the incorporation of viral DNA into the chromosome of the host cell.
Intestinal microbiome: The totality of all DNA sequences of the microorganisms present in the intestine.
Lipopolysaccharides: Main component of the outer cell wall of Gram-negative bacteria.
Lysis: Breaking down of the bacterial cell through damage to the cell membrane.
“Lysis from without”: Lysis of the bacterial cell by binding of phages in excess to the cell surface of the host bacterium.
Lysogenic: Bacteria in whose chromosome a phage genome is integrated are lysogenic.
Lytic: Phages that infect and lyse bacterial cells are lytic.
Microbiome: The totality of all DNA sequences of the microorganisms found in a given environment.
Microbiota: Totality of all microorganisms in a given environment.
Nisin: Antibiotically active peptide from the group of lanthionine-containing antibiotics which is produced by the lactic acid bacterium *Lactococcus lactis*, has a narrow spectrum of activity against certain bacterial species, and is found in raw milk.
Pathogenicity Islands (PAIs): Genetic elements in the bacterial chromosome that code for virulence factors and antibiotic resistance, among other things.
Pediocin: Antibiotically active proteins produced by pediococci, a genus from the family of the *Lactobacillaceae* and inhibit the growth of other bacterial species.
PFU: Plaque forming unit: a phage particle that leads to a circular lysis zone (plaque) in a bacterial lawn.
Phage genome: Totality of all DNA sequences of a phage.
Phageome: Total of all DNA sequences of phages present in a given environment.
Phase variance: Altered transcription products, and therefore proteins, due to repetitive elements in the gene sequence that are either localised in the open reading frame (ORF) of a gene, inside the promoter region or in the vicinity of the promoter. Addition or deletion of the elements results in an altered reading frame for the transcription.
Pilus, *pl*. pili: Filamentous cell extension found in bacteria for attachment to animate or inanimate surfaces.
Porins: Proteins in cell membranes that form pores for mass transfer.
Postharvest sector: After slaughter and harvest
Preharvest sector: Before slaughter and harvest
Prophage: State of a temperate phage when its genome is integrated in the bacterial chromosome.
QPS: Qualified presumption of safety
RBP: Receptor Binding Protein of phages
“ready to eat” (RTE): Food products that are suitable for immediate consumption, offered in refrigerated supermarket display cabinets.
Serotype: A subgroup within a species or strain distinguished by characteristic surface antigens (e.g. O, H, or K antigens); commonly used in diagnostics and epidemiology
Serovar: A taxonomic subdivision within a species defined by differences in antigenic properties; essentially synonymous with “serotype,” but more commonly used in systematic classification
Shigatoxin: Toxins produced by *Shigella dysenteriae* and some *E. coli* strains that result in the death of eukaryotic cells by inhibiting protein synthesis.
spp.: Subspecies
Temperate: Phages are temperate phages when they can both replicate in and lyse the bacterial cell and integrate their genome in the bacterial chromosome, thereby existing as a prophage
Transduction: Transfer of DNA from one bacterial cell to another by phages (generalised transduction: bacterial DNA is unspecifically and randomly packaged into the phage capsid; specialised transduction: DNA sequences adjacent to the integration site of the prophage are packaged into the phage capsid by inaccurate excision of the prophage)
Virus envelope: Protein envelope in which the phage genome and, where applicable, the phage’s own enzymes are packaged
