# Supplementary material for: Limitations and safety aspects related to the use of bacteriophages in food production
Source: FEMS Microbiol Rev. 2026 Jan 20;50:fuag002. doi: 10.1093/femsre/fuag002 (PMC12940142; doi:10.1093/femsre/fuag002)
Supplement: fuag002_Supplemental_File [file fuag002_supplemental_file.pdf]

Supplementary Table S1: Overview of 51 reviews on phage application in the food sector. The topics essential for the use of phages in the food sector are identified by different symbols. (**√:** presented comprehensively; **√:** presented, √: presented briefly or in excerpts; **-** : not subject of the review (*[1]referred as "reduction of pathogens"; *[2]referred as "prevention of spoilage"; *[3]referred as "decontamination of surfaces in animal housing and production facilities"; *[4]single phages or phage mixtures characterised; *[5]phage cocktails, commercially available)

**1. Pre-harvest a) therapy b) bacteria load reduction 2. Post-harvest**			**Biocontrol*[1]**	**Biopreservation*[2]**	**Packaging**	**Biosanitation*[3]**	**Reduction of biofilms**	**Relevant bacterial strains of the food sector**	**Isolated phages*[4] / Phage*[5] cocktails / lytic phage enzymes**	**MOI / efficacy**	**Listed food items: general / detailed**	**Use along production line of food / legal classify-cation of phage use**	**State-ments of: FDA / EFSA**	**Risks / Benefits / risk analysis / research needs**	**Human phage therapy / micro-biom of intestine**	**Biology of phages / History of phage use**	**Reference**
**1. a)**	**1. b)**	**2.**															
√	√	√	√	√	√	√	√	√	√ / √ / -	√ / √	√ / √	√ / -	- / -	√ / √ / - / -	- / -	√ / √	(Amjad et al. 2024)
-	-	√	√	√	√	√	√	√	√ / √ / √	√ / √	√ / -	√ / -	√ / -	√ / √ / √ / √	√ / -	√ / √	(Brovko, Anany and Griffiths 2012)
-	√	√	√	-	-	√	√	√	√ / √ / -	√ / √	√ / √	√ / -	- / -	- / √ / - / -	- / -	√ / -	(Bumunang et al. 2023)
√	√	√	√	√	√	√	√	√	√ / √ / √	- / √	√ / √	√ / -	√ / -	√ / √ / - / -	√ / -	- / √	(Chaudhary et al. 2024)
√	√	√	√	-	√	-	√	√	- / √ / √	√ / √	√ / -	√ / -	√ / -	√ / √ / - / √	- / -	- / √	(Cooper 2016)
√	√	√	√	√	-	√	-	√	√ / √ / -	√ / √	- / -	√ / √	√ / √	- / √ / - / √	- / -	√ / √	(Costa et al. 2023)
√	-	-	√	√	-	√	√	√	- / √ / -	- / √	√ / √	√ / -	- / -	√ / √ / - / -	- / -	√ / √	(Cristobal-Cueto et al. 2021)
√	-	√	√	-	√	-		√	√ / √ / -	- / -	- / -	- / -	√ / -	√ / √ / √ / -	√ / -	- / √	(de Melo, Levesque and Moineau 2018)

1. Pre-harvest a) therapy b) bacteria load reduction 2. Post-harvest: 1. a)	1. b)	2.	Biocontrol*1	Biopreservation*2	Packaging	Biosanitation*3	Reduction of biofilms	Relevant bacterial strains of the food sector	Isolated phages*4 / Phage*5 cocktails / lytic phage enzymes	MOI / efficacy	Listed food items: general / detailed	Use along production line of food / legal classify-cation of phage use	State-ments of: FDA / EFSA	Risks / Benefits / risk analysis / research needs	Human phage therapy / micro-biom of intestine	Biology of phages / History of phage use	Reference
-	-	-	√	-	√	-	√	√	√ / - / √	- / -	- / -	- / -	√ / -	- / - / - / -	√ / -	- / √	(El-Shibiny and El-Sahhar 2017)
√	√	√	√	√	-	√	√	√	√ / √ / -	- / √	√ / -	√ / -	√ / -	√ / √ / √ / -	- / -	√ / √	(Endersen et al. 2014)
√	√	√	√	√	-	-	-	√	√ / √ / √	√ / √	√ / -	√ / -	√ / -	√ / √ / - / -	- / -	- / -	(Endersen and Coffey 2020)
√	√	√	√	√	-	√	-	√	√ / √ / √	- / -	- / -	√ / √	√ / -	√ / √ / - / -	- / -	√ / √	(Fernandez et al. 2018)
-	-	-	√	√	-	-	√	√	- / - / -	- / -	- / √	√ / -	- / -	√ / √ / √ / √	- / -	- / -	(Fister et al. 2019)
√	√	√	√	√	√	√	√	√	- / - / -	√ / √	√	√ / -	√ / -	√ / √ / √ / √	- / -	- / -	(García et al. 2008)
-	-	-	-	-	-	-	-	√	√ / √ / -	√ / √	diary	- / -	- / -	√ / - / - / -	- / -	√ / -	(García-Anaya et al. 2020)
√	√	√	√	√	-	√	√	√	√ / √ / √	- / √	√ / √	√ / -	√ / -	√ / √ / - / -	- / -	- / √	(Garvey 2022)
-	√	√	√	-	-	√	√	√	√ / √ / √	√ / √	√ / √	√ / -	√ / -	√ / √ / √ / -	- / √	- / -	(Ge et al. 2022)
√	√	√	√	-	-	√	√	√	√ / √ / √	- / √	- / -	√ / -	√ / -	√ / √ / - / -	- / -	√ / √	(Gildea, Ayariga and Robertson 2022)
√	√	√	√	-	-	-	-	√	√ / √ / -	√ / √	√ / -	√ / -	√ / -	√ / √ / - / √	- / -	- / -	(Goodridge and Bisha 2011)

1. Pre-harvest a) therapy 1. a)	1. Pre-harvest b) bacteria load reduction 1. b)	2. Post-harvest 2.	Biocontrol*[1]	Biopreservation*[2]	Packaging	Biosanitation*[3]	Reduction of biofilms	Relevant bacterial strains of the food sector	Isolated phages*[4] / Phage*[5] cocktails / lytic phage enzymes	MOI / efficacy	Listed food items: general / detailed	Use along production line of food / legal classify-cation of phage use	State-ments of: FDA / EFSA	Risks / Benefits / risk analysis / research needs	Human phage therapy / micro-biom of intestine	Biology of phages / History of phage use	Reference
√	√	√	√	√	-	-	-	√	- / - / -	- / √	√ / -	√ / -	- / -	√ / √ / √ / √	- / -	- / -	(Greer 2005)
√	√	√	√	√	√	√	√	√	√ / √ / √	√ / √	diary	√ / √	- / -	- / √ / - / -	- / -	- / -	(Gutiérrez et al. 2019)
-	√	√	√	-	-	-	-	√	√ / √ / -	√ / √	√ / √	√ / -	√ / -	√ / √ / √ / -	- / -	√ / -	(Hagens and Loessner 2010)
√	-	√	√	-	-	-	√	√	√ / √ / -	- / √	√ / √	√ / -	√ / -	√ / √ / √ / √	√ / √	√ / √	(Hassan et al. 2021)
-	√	√	√	√	-	√	√	√	√ / √ /√	√ / √	- / -	√ / -	- / -	√ / √ / √ / √	- / -	√ / √	(Hudson et al. 2005)
-	-	-	√	-	√		√	√	√ / √ / -	√ / √	√ / √	√ / -	√ / √	√ / √ / - / -	- / -	√ / √	(Hyla, Dusza and Skaradzińska 2022)
-	-	√	√	-	√	√	√	√	√ / √ / √	√ / √	√ / -	- / -	√ / -	- / - / √ / √	- / -	√ / √	(Imran et al. 2023)
-	√	√	√	√	-	√	√	√	√ / √ / -	√ / √	√ / √	√ / -	√ / -	√ / √ / - / -	- / -	√ / √	(Jagannathan, Dakoske and Vijayakumar 2022)
√	√	√	√	√	√	√	√	√	√ / √ / √	- / √	- / -	- / -	√ / -	√ / √ / -/ √	√ / √	√ / √	(Jaglan et al. 2022)

1. Pre-harvest a) therapy b) bacteria load reduction 2. Post-harvest			Biocontrol*[1]	Biopreservation*[2]	Packaging	Biosanitation*[3]	Reduction of biofilms	Relevant bacterial strains of the food sector	Isolated phages*[4] / Phage*[5] cocktails / lytic phage enzymes	MOI / efficacy	Listed food items: general / detailed	Use along production line of food / legal classify-cation of phage use	State-ments of: FDA / EFSA	Risks / Benefits / risk analysis / research needs	Human phage therapy / micro-biom of intestine	Biology of phages / History of phage use	Reference
1. a)	1. b)	2.															
√	√	√	√	√	-	√	√	√	√ / √ / √	√ / √	√ / √	√ / -	- / -	- / √ / - / -	- / -	√ / -	(Kazi and Annapure 2016)
√	√	√	√	-	-	-	√	√	√ / √ / -	√ / √	√ / √	√ / √	- / -	√ / √ / √ / -	- / -	√ / -	(Lavilla et al. 2023)
-	-	-	√	-	√	-	√	√	- / √ / √	- / √	√ / -	- / -	√ / -	√ / √ / - / -	- / -	√ / √	(Lee, Kim and Ryu 2023)
√	√	√	√	√	-	-	√	√	√ / √ / -	√ / √	√ / √	- / -	√ / -	√ / √ / √ / -	- / -	√ / -	(Lewis and Hill 2020)
√	√	√	√	√	√	√	√	√	- / √ / -	- / √	√ / √	√ / -	√ / √	√ / √ / √ / -	- / -	- / -	(Li et al. 2022)
-	-	-	-	-	-	-	-	√	- / √ / -	√ / √	√ / -	- / -	√ / -	- / - / - / -	- / -	√ / √	(Liu, Quek and Huang 2023)
√	√	√	√	√	√	√	√	√	√ / √ / √	√ / √	√ / √	√ / -	√ / -	√ / √ / - / -	√ / √	√ / -	(Mills, Ross and Hill 2017)
-	√	√	√	-	√	-	-	√	√ / √ / -	√ / √	√ / √	√ / -	√ / -	√ / √ / - / -	- / -	- / -	(Moye, Woolston and Sulakvelidze 2018)
√	√	√	√	√	√	√	√	√	√ / √ / √	- / √	√ / √	√ / -	√ / -	- / √ / - / -	- / -	√ / √	(O'Sullivan et al. 2019)

1. Pre-harvest a) therapy b) bacteria load reduction 2. Post-harvest: 1. a)	1. b)	2.	Biocontrol*1	Biopreservation*2	Packaging	Biosanitation*3	Reduction of biofilms	Relevant bacterial strains of the food sector	Isolated phages*4 / Phage*5 cocktails / lytic phage enzymes	MOI / efficacy	Listed food items: general / detailed	Use along production line of food / legal classify-cation of phage use	State-ments of: FDA / EFSA	Risks / Benefits / risk analysis / research needs	Human phage therapy / micro-biom of intestine	Biology of phages / History of phage use	Reference
-	-	√	√	√	-	√	√	√	√ / √ / -	√ / √	√ / √	- / -	√ / -	- / √ / - / -	- / -	- / -	(Pérez Pulido et al. 2016)
-	-	-	√	-	-	-	-	*E.coli*	- / √ / -	√ / √	√ / -	- / -	√ / √	√ / √ / -/ -	- / √	√ / -	(Pinto, Almeida and Azeredo 2020)
√	√	√	√	√	√	√	√	√	√ / √ / √	- / √	√ / √	√ / -	√ / √	√ / √ / -/ -	- / -	- / -	(Połaska and Sokołowska 2019)
-	-	√	√	√	√	√	√	√	√ / √ / √	- / √	**diary**	√ / -	√ / √	√ / √ / -/ -	- / -	√ / -	(Poonia, Mishra and Rai 2020)
-	-	-	√	√	√	√	√	√	√ / √ / √	- / -	**diary**	- / -	- / -	- / √ / - / -	- / -	√ / -	(Pujato, Quiberoni and Mercanti 2019)
√	√	√	√	√	√	√	√	√	√ / √ / √	- / -	√ / √	√ / -	- / -	√ / √ / √ / -	- / -	√ / -	(Ramos-Vivas et al. 2021)
√	√	√	√	√	-	√	√	√	√ / √ / √	- / √	√ / √	√ / -	- / -	√ / √ / -/ -	√ / √	√ / √	(Ranveer et al. 2024)
√	√	√	√	-	√	√	√	√	- / √ / -	- / -	√ / √	√ / -	√ / -	√ / √ / √ / -	√ / -	√ / √	(Sarhan and Azzazy 2015)
√	√	√	√	√	√	√	√	√	√ / √ / -	√/ √	√ / √	√ / -	√ / -	√ / √ / - / -	- / -	- / -	(Sillankorva, Oliveira and Azeredo 2012)

1. Pre-harvest a) therapy b) bacteria load reduction 2. Post-harvest			Biocontrol*1	Biopreservation*2	Packaging	Biosanitation*3	Reduction of biofilms	Relevant bacterial strains of the food sector	Isolated phages*4 / Phage*5 cocktails / lytic phage enzymes	MOI / efficacy	Listed food items: general / detailed	Use along production line of food / legal classify-cation of phage use	State-ments of: FDA / EFSA	Risks / Benefits / risk analysis / research needs	Human phage therapy / micro-biom of intestine	Biology of phages / History of phage use	Reference
1. a)	1. b)	2.															
-	√	√	√	-	-	√	-	√	√ / √ / -	- / √	√ / √	- / √	√ / -	√ / √ / -/ -	- / -	- / -	(Sulakvelidze 2013)
√	√	√	√	-	-	-	√	√	- / √ / -	- / √	√ / √	√ / -	√ / -	√ / √ / √ / -	- / -	√ / √	(Tan, Chan and Lee 2014)
-	√	√	√	-	√	√	√	√	√ / √ / -	√ / √	√ / √	√ / -	√ / -	√ / √ / - / -	- / -	- / √	(Vikram, Woolston and Sulakvelidze 2021)
-	√	√	√	√	√	√	√	√	√ / √ / -	√ / √	√ / -	√ / -	√ / -	√ / √ / - / -	- / -	√ / -	(Wang and Zhao 2022)
√	√	√	√	√	√	√	√	√	-√ / √ / √	- / √	√ / √	√ / -	√ / -	√ / √ / -/ -	- / -	- / -	(Xu 2021)

References:

Amjad N, Naseer MS, Imran A et al. A mini-review on the role of bacteriophages in food safety. *CyTA-J Food* 2024;**22**:e2357192. https://doi.org/10.1080/19476337.2024.2357192

Brovko LY, Anany H, Griffiths MW. Bacteriophages for detection and control of bacterial pathogens in food and food-processing environment. *Adv Food Nutr Res* 2012;**67**:241-88. https://doi.org/10.1016/b978-0-12-394598-3.00006-x

Bumunang EW, Zaheer R, Niu D et al. Bacteriophages for the targeted control of foodborne pathogens. *Foods* 2023;**12**:2734. https://doi.org/10.3390/foods12132734

Chaudhary V, Kajla P, Lather D et al. Bacteriophages: a potential game changer in food processing industry. *Crit Rev Biotechnol* 2024;**44**:1325-49. https://doi.org/10.1080/07388551.2023.2299768

Cooper IR. A review of current methods using bacteriophages in live animals, food and animal products intended for human consumption. *J Microbiol Methods* 2016;**130**:38-47. https://doi.org/10.1016/j.mimet.2016.07.027
Costa MJ, Pastrana LM, Teixeira JA et al. Bacteriophage delivery systems for food applications: opportunities and perspectives. *Viruses* 2023;**15**:1271. https://doi.org/10.3390/v15061271
Cristobal-Cueto P, García-Quintanilla A, Esteban J et al. Phages in food industry biocontrol and bioremediation. *Antibiotics* 2021;**10**:786. https://doi.org/10.3390/antibiotics10070786
de Melo AG, Levesque S, Moineau S. Phages as friends and enemies in food processing. *Curr Opin Biotechnol* 2018;**49**:185-90. https://doi.org/10.1016/j.copbio.2017.09.004
El-Shibiny A, El-Sahhar S. Bacteriophages: the possible solution to treat infections caused by pathogenic bacteria. *Can J Microbiol* 2017;**63**:865-79. https://doi.org/10.1139/cjm-2017-0030
Endersen L, apos, Mahony J et al. Phage therapy in the food industry. *Annu Rev Food Sci Technol* 2014;**5**:327-49. https://doi.org/10.1146/annurev-food-030713-092415
Endersen L, Coffey A. The use of bacteriophages for food safety. *Curr Opin Food Sci* 2020;**36**:1-8. https://doi.org/10.1016/j.cofs.2020.10.006
Fernandez L, Gutierrez D, Rodriguez A et al. Application of bacteriophages in the agro-food sector: a long way toward approval. *Front Cell Infect Microbiol* 2018;**8**:296. https://doi.org/10.3389/fcimb.2018.00296
Fister S, Mester P, Witte AK et al. Part of the problem or the solution? Indiscriminate use of bacteriophages in the food industry can reduce their potential and impair growth-based detection methods. *Trends Food Sci Technol* 2019;**90**:170-4. https://doi.org/10.1016/j.tifs.2019.02.031
García-Anaya MC, Sepulveda DR, Sáenz-Mendoza AI et al. Phages as biocontrol agents in dairy products. *Trends Food Sci Technol* 2020;**95**:10-20. https://doi.org/https://doi.org/10.1016/j.tifs.2019.10.006
García P, Martínez B, Obeso JM et al. Bacteriophages and their application in food safety. *Lett Appl Microbiol* 2008;**47**:479-85. https://doi.org/10.1111/j.1472-765X.2008.02458.x
Garvey M. Bacteriophages and food production: biocontrol and bio-preservation options for food safety. *Antibiotics* 2022;**11**:1324. https://doi.org/10.3390/antibiotics11101324
Ge H, Fu S, Guo H et al. Application and challenge of bacteriophage in the food protection. *Int J Food Microbiol* 2022;**380**:109872. https://doi.org/10.1016/j.ijfoodmicro.2022.109872
Gildea L, Ayariga JA, Robertson BK. Bacteriophages as biocontrol agents in livestock food production. *Microorganisms* 2022;**10**:2126. https://doi.org/10.3390/microorganisms10112126
Goodridge LD, Bisha B. Phage-based biocontrol strategies to reduce foodborne pathogens in foods. *Bacteriophage* 2011;**1**:130-7. https://doi.org/10.4161/bact.1.3.17629
Greer GG. Bacteriophage control of foodborne bacteriat. *J Food Prot* 2005;**68**:1102-11. https://doi.org/10.4315/0362-028x-68.5.1102
Gutiérrez D, Fernández L, Rodríguez A et al. Role of bacteriophages in the implementation of a sustainable dairy chain. *Front Microbiol* 2019;**10**:12. https://doi.org/10.3389/fmicb.2019.00012

Hagens S, Loessner MJ. Bacteriophage for biocontrol of foodborne pathogens: calculations and considerations. *Curr Pharm Biotechnol* 2010;**11**:58-68. https://doi.org/10.2174/138920110790725429

Hassan AY, Lin JT, Ricker N et al. The age of phage: friend or foe in the new dawn of therapeutic and biocontrol applications? *Pharmaceuticals* 2021;**14**:199. https://doi.org/10.3390/ph14030199

Hudson JA, Billington C, Carey-Smith G et al. Bacteriophages as biocontrol agents in food. *J Food Prot* 2005;**68**:426-37. https://doi.org/10.4315/0362-028X-68.2.426

Hyla K, Dusza I, Skaradzińska A. Recent advances in the application of bacteriophages against common foodborne pathogens. *Antibiotics* 2022;**11**. https://doi.org/10.3390/antibiotics11111536

Imran A, Shehzadi U, Islam F et al. Bacteriophages and food safety: an updated overview. *Food Sci Nutr* 2023;**11**:3621-30. https://doi.org/10.1002/fsn3.3360

Jagannathan BV, Dakoske M, Vijayakumar PP. Bacteriophage-mediated control of pre- and post-harvest produce quality and safety. *LWT* 2022;**169**:113912. https://doi.org/10.1016/j.lwt.2022.113912

Jaglan AB, Anand T, Verma R et al. Tracking the phage trends: a comprehensive review of applications in therapy and food production. *Front Microbiol* 2022;**13**:993990. https://doi.org/10.3389/fmicb.2022.993990

Kazi M, Annapure US. Bacteriophage biocontrol of foodborne pathogens. *J Food Sci Technol* 2016;**53**:1355-62. https://doi.org/10.1007/s13197-015-1996-8

Lavilla M, Domingo-Calap P, Sevilla-Navarro S et al. Natural killers: opportunities and challenges for the use of bacteriophages in microbial food safety from the one health perspective. *Foods* 2023;**12**:552. https://doi.org/10.3390/foods12030552

Lee C, Kim H, Ryu S. Bacteriophage and endolysin engineering for biocontrol of food pathogens/pathogens in the food: recent advances and future trends. *Crit Rev Food Sci Nutr* 2023;**63**:8919-38. https://doi.org/10.1080/10408398.2022.2059442

Lewis R, Hill C. Overcoming barriers to phage application in food and feed. *Curr Opin Biotechnol* 2020;**61**:38-44. https://doi.org/10.1016/j.copbio.2019.09.018

Li J, Zhao F, Zhan W et al. Challenges for the application of bacteriophages as effective antibacterial agents in the food industry. *J Sci Food Agric* 2022;**102**:461-71. https://doi.org/10.1002/jsfa.11505

Liu S, Quek S-Y, Huang K. Advanced strategies to overcome the challenges of bacteriophage-based antimicrobial treatments in food and agricultural systems. *Crit Rev Food Sci Nutr* 2023;**64**:12574-98. https://doi.org/10.1080/10408398.2023.2254837

Mills S, Ross RP, Hill C. Bacteriocins and bacteriophage; a narrow-minded approach to food and gut microbiology. *FEMS Microbiol Rev* 2017;**41**:S129-53. https://doi.org/10.1093/femsre/fux022

Moye ZD, Woolston J, Sulakvelidze A. Bacteriophage applications for food production and processing. *Viruses* 2018;**10**:205. https://doi.org/10.3390/v10040205

O'Sullivan L, Bolton D, McAuliffe O et al. Bacteriophages in food applications: from foe to friend. *Annu Rev Food Sci Technol* 2019;**10**:151-72. https://doi.org/10.1146/annurev-food-032818-121747

Pérez Pulido R, Grande Burgos MJ, Gálvez A et al. Application of bacteriophages in post-harvest control of human pathogenic and food spoiling bacteria. *Crit Rev Biotechnol* 2016;**36**:851-61. https://doi.org/10.3109/07388551.2015.1049935

Pinto G, Almeida C, Azeredo J. Bacteriophages to control shiga toxin-producing *E. coli* - safety and regulatory challenges. *Crit Rev Biotechnol* 2020;**40**:1081-97. https://doi.org/10.1080/07388551.2020.1805719

Połaska M, Sokołowska B. Bacteriophages-a new hope or a huge problem in the food industry. *AIMS Microbiol* 2019;**5**:324-46. https://doi.org/10.3934/microbiol.2019.4.324

Poonia A, Mishra A, Rai C. Scope and Applications of Bacteriophage in Dairy Industry. *ResearchGate* 2020. http://www.researchgate.net/publication/348275436_Scope_and_Applications_of_Bacteriophage_in_Dairy_Industry

Pujato SA, Quiberoni A, Mercanti DJ. Bacteriophages on dairy foods. *J Appl Microbiol* 2019;**126**:14-30. https://doi.org/10.1111/jam.14062

Ramos-Vivas J, Elexpuru-Zabaleta M, Samano ML et al. Phages and enzybiotics in food biopreservation. *Molecules* 2021;**26**:5138. http://https://www.mdpi.com/1420-3049/26/17/5138

Ranveer SA, Dasriya V, Ahmad MF et al. Positive and negative aspects of bacteriophages and their immense role in the food chain. *NPJ Sci Food* 2024;**8**:1. https://doi.org/10.1038/s41538-023-00245-8

Sarhan WA, Azzazy HM. Phage approved in food, why not as a therapeutic? *Expert Rev Anti Infect Ther* 2015;**13**:91-101. https://doi.org/10.1586/14787210.2015.990383

Sillankorva SM, Oliveira H, Azeredo J. Bacteriophages and their role in food safety. *Int J Microbiol* 2012;**2012**:863945. https://doi.org/10.1155/2012/863945

Sulakvelidze A. Using lytic bacteriophages to eliminate or significantly reduce contamination of food by foodborne bacterial pathogens. *J Sci Food Agric* 2013;**93**:3137-46. https://doi.org/10.1002/jsfa.6222

Tan T, Chan K, Lee L. Application of bacteriophage in biocontrol of major foodborne bacterial pathogens. *J Mol Biol Mol Imaging* 2014;**1**:9. http://www.austinpublishinggroup.com/molecular-biology/fulltext/jmbmi-v1-id1004.pdf

Vikram A, Woolston J, Sulakvelidze A. Phage biocontrol applications in food production and processing. *Curr Issues Mol Biol* 2021;**40**:267-302. https://doi.org/10.21775/cimb.040.267

Wang Z, Zhao X. The application and research progress of bacteriophages in food safety. *J Appl Microbiol* 2022;**133**:2137-47. https://doi.org/10.1111/jam.15555

Xu Y. Phage and phage lysins: new era of bio-preservatives and food safety agents. *J Food Sci* 2021;**86**:3349-73. https://doi.org/10.1111/1750-3841.15843
